# Relative assembly maps and the *K*-theory of Hecke algebras in prime characteristic

**DOI:** 10.1007/s00208-024-02966-x

**Published:** 2024-08-23

**Authors:** W. Lück

**Affiliations:** Mathematicians Institut der Universität Bonn, Endenicher Allee 60, 53115 Bonn, Germany

**Keywords:** 19D35, 19D50, 20C08

## Abstract

We investigate the relative assembly map from the family of finite subgroups to the family of virtually cyclic subgroups for the algebraic *K*-theory of twisted group rings of a group *G* with coefficients in a regular ring *R* or, more generally, with coefficients in a regular additive category. They are known to be isomorphisms rationally. We show that it suffices to invert only those primes *p* for which *G* contains a non-trivial finite *p*-group and *p* is not invertible in *R*. The key ingredient is the detection of Nil-terms of a twisted group ring of a finite group *F* after localizing at *p* in terms of the *p*-subgroups of *F* using Verschiebungs and Frobenius operators. We construct and exploit the structure of a module over the ring of big Witt vectors on the Nil-terms. We analyze the algebraic *K*-theory of the Hecke algebras of subgroups of reductive *p*-adic groups in prime characteristic.

## Introduction

We first state and discuss the main results of this paper. In this introduction we only consider rings as coefficients for simplicity. Many of the results will extend to additive categories as coefficients. Moreover, in all cases one can allow a twisting by a *G*-action on *R* or a *G*-action on the additive category. Groups are understood to be discrete, unless explicitly stated otherwise.

### On the *K*-theoretic relative assembly map from ${{{\mathcal {F}}}\textrm{in}}$ to ${{{\mathcal {V}}}\textrm{cyc}}$ for regular coefficient rings

If $\mathcal {P}$ is a set of primes and $f :A \rightarrow B$ is a homomorphisms of abelian groups, we call *f* a $\mathcal {P}$-*isomorphism* if the map ${{\,\textrm{id}\,}}_{\mathbb {Z}[\mathcal {P}^{-1}]} \otimes _ {\mathbb {Z}} f :\mathbb {Z}[\mathcal {P}^{-1}] \otimes _{\mathbb {Z}} A \rightarrow \mathbb {Z}[\mathcal {P}^{-1}] \otimes _{\mathbb {Z}} B$ is bijective, where the ring $\mathbb {Z}[\mathcal {P}^{-1}]$ satisfies $\mathbb {Z}\subseteq \mathbb {Z}[\mathcal {P}^{-1}] \subseteq \mathbb {Q}$ and is obtained from $\mathbb {Z}$ by inverting all primes in $\mathcal {P}$.

#### Notation 1.1

For a group *G* and a ring *R*, let $\mathcal {P}(G,R)$ be the set of primes, which are not invertible in *R* and for which *G* contains a non-trivial finite *p*-subgroup.

If *G* is torsionfree or, more generally, the order of any finite subgroup of *G* is invertible in *R*, then $\mathcal {P}(G,R)$ is empty and $\mathcal {P}$-isomorphism means just isomorphism. If $R = \mathbb {Z}$, then $\mathcal {P}(G,\mathbb {Z})$ is the set of primes *p* for which *G* contains an element of order *p*.

#### Theorem 1.2

Let *R* be a regular ring coming with a group homomorphism $\rho :G \rightarrow {{\,\textrm{aut}\,}}(R)$ to the group of ring automorphisms of *R*. Then the relative assembly map

$$\begin{aligned} H_n^G(E_{{{{\mathcal {F}}}\textrm{in}}}(G);\textbf{K}_R) \rightarrow H_n^G(E_{{{{\mathcal {V}}}\textrm{cyc}}}(G);\textbf{K}_R) \end{aligned}$$

is a $\mathcal {P}(G,R)$-isomorphism for all $n \in \mathbb {Z}$.

Theorem 1.2 improves [28, Theorem 0.3], where $\mathcal {P}(G,R)$ is required to be the set of all primes. See also [20, Theorem 5.11] for $R = \mathbb {Z}$. If the order of any finite subgroup of *G* is invertible in *R* and the action $\rho$ is trivial, then Theorem 1.2 has already been proved in [26, Proposition 2.6 on page 686]. Theorem 1.2 is a special case of Theorem 9.1. Note that the relative assembly map appearing in Theorem 1.2 is always split injective, see [10, Theorem 1.3], [28, Theorem 0.1].

For more information about the relative assembly map appearing in Theorem 1.2 and the *K*-theoretic Farrell–Jones Conjecture, we refer to Remark 6.4 and [25].

The *subgroup category of* *G* *for the family* ${{{\mathcal {F}}}\textrm{in}}$ *of finite subgroups*  has as objects finite subgroups *H* of *G*. For finite subgroups *H* and *K* of *G*, denote by ${{\,\textrm{conhom}\,}}_G(H,K)$ the set of group homomorphisms $f:H \rightarrow K$, for which there exists an element $g \in G$ with $gHg^{-1} \subset K$ such that *f* is given by conjugation with *g*, i.e. $f = c(g): H \rightarrow K, h \mapsto ghg^{-1}$. Note that $c(g) = c(g')$ holds for two elements $g,g' \in G$ with $gHg^{-1} \subset K$ and $g'Hg'^{-1} \subset K$ if and only if $g^{-1}g'$ lies in the centralizer $C_GH = \{g \in G \mid gh=hg \text{ for } \text{ all } h \in H\}$ of *H* in *G*. The group of inner automorphisms ${\text {Inn}}(K)$ of *K* acts on ${{\,\textrm{conhom}\,}}_G(H,K)$ from the left by composition. Define the set of morphismsEquivalently,  is the double coset $K \backslash \{ g \in G \mid gHg^{-1} \subseteq K\} / C_G(H)$ where the left *K*-action and the right $C_G(H)$-action come from the multiplication in *G*.

#### Remark 1.3

(*The Full Farrell–Jones Conjecture*) The Full Farrell–Jones Conjecture is stated in [25, Conjecture 13.27]. Here we only need to know that it implies that the assembly map

1.4 $$\begin{aligned} H_n^G(E_{{{{\mathcal {V}}}\textrm{cyc}}}(G);\textbf{K}_R) \rightarrow K_n(R_{\rho }[G]) \end{aligned}$$

is bijective for all $n \in \mathbb {Z}$ and any ring *R* coming with a group homomorphism $\rho :G \rightarrow {{\,\textrm{aut}\,}}(R)$, see [25, Theorem 13.61 (i)].

Note that the Full Farrell–Jones Conjecture is known to be true for a large class of groups including hyperbolic groups, CAT(0)-groups, lattices in locally compact second countable Hausdorff groups, and fundamental groups of manifolds of dimension $\le 3$ and has useful inheritance properties, e.g., passing to subgroups and overgroups of finite index, see for instance [25, Chapter 15].

#### Theorem 1.5

Suppose *G* satisfies the Full Farrell–Jones Conjecture, Let *R* be a regular ring coming with a group homomorphism $\rho :G \rightarrow {{\,\textrm{aut}\,}}(R)$ such that the order of any finite subgroup of *G* is invertible in *R*.

Then the canonical mapis an isomorphism and

$$\begin{aligned} K_n(R_{\rho }[G]) = 0 \quad \text {for} \;n \le -1. \end{aligned}$$

where $R_{\rho }[G]$ denotes the $\rho$-twisted group ring.

### On the *K*-theoretic relative assembly map from ${{{\mathcal {F}}}\textrm{in}}$ to ${{{\mathcal {V}}}\textrm{cyc}}$ for Artinian coefficient rings

In the case that *R* is an Artinian ring, we get even an integral result in degree $n \le 0$ without the assumption that the order of any finite subgroup of *G* is invertible in *R*.

#### Theorem 1.6

Let *G* be a discrete group which satisfies the Full Farrell–Jones Conjecture. Let *R* be an Artinian ring coming with a group homomorphism $\rho :G \rightarrow {{\,\textrm{aut}\,}}(R)$.

Then the canoncial mapis an isomorphism and

$$\begin{aligned} K_n(R_{\rho }[G]) = 0 \quad \text {for} \;n \le -1. \end{aligned}$$

#### Proof

This is proved in [25, Theorem 13.61 (v)] for trivial $\rho$. It directly extends to the case where $\rho$ is non-trivial. $\square$

Note that the prototype of results such as Theorem 1.6 is due to Moody [31], who proved the bijectivity of the canonical map  for *R* a field of characteristic zero and *G* a virtually polycyclic group.

### On the *K*-theoretic relative assembly map from ${{{\mathcal {F}}}\textrm{in}}$ to ${{{\mathcal {V}}}\textrm{cyc}}$ for fields as coefficients

For the reader’s convenience we summarize what happens in the special case, where *R* is a skew-field *F*. Note that a skew-field of characteristic zero is regular and satisfies $\mathcal {P}(G,F) = \emptyset$ and any skew-field is an Artinian ring. Hence we conclude from Theorems 1.2, 1.5, and 1.6

#### Theorem 1.7

Let *G* be a group and *F* be a skew-field coming with a group homomorphism $\rho :G \rightarrow {{\,\textrm{aut}\,}}(F)$ into the group of field automorphism of *F*. Then:

(i) The relative assembly map
  $$\begin{aligned} H_n^G(E_{{{{\mathcal {F}}}\textrm{in}}}(G);\textbf{K}_F) \rightarrow H_n^G(E_{{{{\mathcal {V}}}\textrm{cyc}}}(G);\textbf{K}_F) \end{aligned}$$
  is bijective for every $n \in \mathbb {Z}$ if one of the following condition holds:
  *F* has characteristic zero;
  There exists a prime *p* such that *F* has characteristic *p* and *G* contains no non-trivial finite *p*-group;
(ii) If *p* is a prime and *F* is a skew-field of characteristic *p*, then the map
  $$\begin{aligned} H_n^G(E_{{{{\mathcal {F}}}\textrm{in}}}(G);\textbf{K}_F)[1/p]\rightarrow H_n^G(E_{{{{\mathcal {V}}}\textrm{cyc}}}(G);\textbf{K}_F)[1/p] \end{aligned}$$
  is bijective for every $n \in \mathbb {Z}$;
(iii) The canonical map  is bijective if *G* satisfies the Full Farrell–Jones Conjecture;
(iv) We have $K_n(F_{\rho }[G])$ for $n \le -1$, if *G* satisfies the Full Farrell–Jones Conjecture.

### On the *K*-theoretic relative assembly map from ${{{\mathcal {F}}}\textrm{in}}$ to ${{{\mathcal {V}}}\textrm{cyc}}$ for $\mathbb {Z}$ as coefficients

For the reader’s convenience we summarize what is known in the special case where *R* is the ring $\mathbb {Z}$ of integers.

#### Theorem 1.8

Let *G* be a group. Then

(i) The relative assembly map
  $$\begin{aligned} H_n^G(E_{{{{\mathcal {F}}}\textrm{in}}}(G);\textbf{K}_\mathbb {Z}) \rightarrow H_n^G(E_{{{{\mathcal {V}}}\textrm{cyc}}}(G);\textbf{K}_\mathbb {Z}) \end{aligned}$$
  is a $\mathcal {P}(G;\mathbb {Z})$-isomorphism for every $n \in \mathbb {Z}$, where $\mathcal {P}(G,\mathbb {Z})$ is the set of primes *p*, for which *G* contains a non-trivial finite *p*-group;
(ii) The canonical map  is bijective and $K_n(\mathbb {Z}[G]) = 0$ for $n \le -2$ if *G* satisfies the Full Farrell–Jones Conjecture;
(iii) Suppose that *G* is torsionfree and satisfies the Full Farrell–Jones Conjecture. Then $K_n(\mathbb {Z}G)$ for $n \le -1$, the reduced projective class group $\widetilde{K}_0(\mathbb {Z}G)$, and the Whitehead group ${{\,\textrm{Wh}\,}}(G)$ are trivial.

#### Proof

(i) This follows directly from Theorem 1.2.

(ii) See [25, Theorem 13.61 (vi)].

(iii) See [25, Theorem 13.61 (iii) and (iv)]. $\square$

### Totally disconnected groups

So far *G* has been a discrete group. Now we want to deal with td-groups, i.e., locally compact second countable totally disconnected topological Hausdorff groups, and the algebraic *K*-theory of their Hecke algebras. In some special cases or in a weaker form, we extend the main results of [5] from characteristic zero to prime characteristic.

Let *R* be a (not necessarily commutative) ring. We will need the following assumption to make sense of the notion of a Hecke algebra. It is taken from [14, page 9].

#### Assumption 1.9

There exists a compact open subgroup $\widetilde{U}$ of *G* such that for any compact open subgroup $\widetilde{U}'\subseteq \widetilde{U}$ of $\widetilde{U}$ the index $[\widetilde{U}: \widetilde{U}']$ is invertible in *R*.

This assumption is automatically satisfied if *G* is discrete, since then we can take $\widetilde{U} = \{1\}$, or if $\mathbb {Q}\subseteq R$. If *p* is a prime number which is invertible in *R*, then Assumption 1.9 is satisfied for any subgroup of a reductive *p*-adic group *G* by [30, Lemma 1.1] and Lemma 11.2.

Suppose that Assumption 1.9 is satisfied. Then the Hecke algebra $\mathcal {H}(G;R)$ is defined as the algebra of locally constant functions $G \rightarrow R$ with compact support and multiplication given by convolution, see for instance [7, Section 11].

One may consider the *K*-groups $K_n(\mathcal {H}(G;R))$ of $\mathcal {H}(G;R)$ for $n \in \mathbb {Z}$. There is an assembly map1.10Here $H_*^G$ is a *G*-homology theory digesting smooth *G*-*CW*-complexes, which satisfies $\mathcal {H}^G_n(G/U;\textbf{K}_R) \cong K_n(\mathcal {H}(U;R))$ for every open subgroup *U* of *G*, the *G*-*CW*-complex  is any model for the classifying space for proper smooth *G*-actions, and the map (1.10) is induced by the projection . We say that *R* is *l-uniformly regular* for the natural number *l* if *R* is noetherian and every *R*-module admits a projective resolution of length at most *l*. We call *R* *uniformly regular* if *R* is *l*-uniformly regular for some natural number *l*. If *G* is modulo a compact subgroup isomorphic to a closed subgroup of a reductive *p*-adic group and *R* is uniformly regular and satisfies $\mathbb {Q}\subseteq R$, e.g., *R* is a field of characteristic zero, then we conclude from [5, Corollary 1.18] that the map (1.10) is bijective, $K_n(\mathcal {H}(G;R))$ vanishes for $n \le -1$, and the canonical map1.11is bijective. Here  is analogously defined as  but now for  the family of compact open subgroups of *G*.

Next we want to explain what we can say in the case, where the condition that *R* is uniformly regular and satisfies $\mathbb {Q}\subseteq R$ is weakened to the condition that *R* is uniformly regular and only certain primes have to be invertible in *R* or to the condition that $N \cdot 1_R = 0$ holds in *R* for some natural number *N*.

#### Theorem 1.12

Let *p* be a prime. Assume that *G* is modulo a compact subgroup isomorphic to a closed subgroup of a reductive *p*-adic group. Let *N* be a natural number and let *R* a be ring with unit $1_R$.

(i) Suppose that $N \cdot 1_R = 0$ and that Assumption 1.9 is satisfied. Then the assembly map (1.10) induces an isomorphism  for every $n \in \mathbb {Z}$;
(ii) Suppose that $N \cdot 1_R = 0$, and that *R* is Artinian, e.g., *R* is a field of prime characteristic *q* for $p \not = q$ and we take $N = q$. Suppose that Assumption 1.9 is satisfied. Then
  $$\begin{aligned} K_n (\mathcal {H}(G;R))[1/N] = 0 \quad \text {for}\; n \le -1 \end{aligned}$$
  and the map induced by (1.11)  is bijective;
(iii) Suppose that *R* is uniformly regular and for any two compact open subgroups $U_0$ and $U_1$ of *G* with $U_0 \subseteq U_1$ the index $[U_1: U_0]$ is invertible in *R*. Then:

- Assumption 1.9 is satisfied;
- The map (1.10) is bijective for all $n \in \mathbb {Z}$;
- We have $K_n (\mathcal {H}(G;R))= 0$ for $n \le 1$;
- The canoncial map  is bijective.

One can define more general Hecke algebras $\mathcal {H}(G,R,\rho ,\omega )$ allowing a *G*-action $\rho$ on *R* and central character $\omega$ and everything carries over to this more general setting, see Remark 11.6.

The proof of Theorem 1.12 will be given in Sect. 11.4 and is based on a version of the Farrell–Jones Conjecture for totally disconnected groups with categories with *G*-support as coefficients.

### On the twisted Nil-terms of finite groups

The proof of some of the results above relies on the following theorem.

Let *F* be a finite group and $\alpha :F \rightarrow F$ be a group automorphism. Let $F \rtimes _{\alpha } \mathbb {Z}$ be the semidirect product associated to $\alpha$, where for the standard generator $t \in \mathbb {Z}$ we have $tft^{-1} = \alpha (f)$ for $f \in F$. Let *R* be a ring coming with a group homomorphism $\mu :F \rtimes _{\alpha } \mathbb {Z}\rightarrow {{\,\textrm{aut}\,}}(R)$. Let $\rho :F \rightarrow {{\,\textrm{aut}\,}}(R)$ be the restriction of $\mu$ to *F*. Our goal is to get information about the structure of the Nil-groups

$$\begin{aligned} N\!K_n(R_{\rho }[F],\Psi ) = \overline{K}_{n-1}({{\,\textrm{Nil}\,}}(R_{\rho }[F],\Psi )) \end{aligned}$$

with respect to the ring automorphism $\psi :R_{\rho }[F] \xrightarrow {\cong } R_{\rho }[F]$ sending $r\cdot f$ for $r \in R$ and $f \in F$ to $\mu (t)(r) \cdot \alpha (f)$, where $\overline{K}_{n-1}({{\,\textrm{Nil}\,}}(R_{\rho }[F],\Psi ))$ is defined in Notation 4.6 taking $\mathcal {A}= \underline{R_{\rho }[F]}$ for the $\mathbb {Z}$-category $\underline{R_{\rho }[F]}$, which has precisely one object and whose $\mathbb {Z}$-module of endomorphisms is $R_{\rho }[F]$. These Nil-groups appear in the twisted Bass-Heller-Swan decomposition for $R_{\mu }[F \rtimes _{\alpha }\mathbb {Z}] = (R_{\rho }[F])_{\psi }[\mathbb {Z}]$, see (4.5).

Fix a prime number *p*. Let $T_p$ be the set of triples (*P*, *k*, *y*) consisting of a *p*-subgroup *P* of *F*, an integer *k* with $k \ge 1$, and an element $y \in F$ such that $c_y \circ \alpha ^k(P) = P$ holds for the automorphism $c_y :F \rightarrow F$ sending *z* to $yzy^{-1}$. Let $\psi _{(P,k,y)} :R_{\rho |_P}[P] \xrightarrow {\cong } R_{\rho |_P}[P]$ be the ring automorphism sending $r \cdot p$ for $r \in R$ and $p \in P$ to $\mu (yt^k)(r) \cdot c_y \circ \alpha ^k(p)$.

Given a triple $(P,k,y) \in T_p$, define a functor of Nil-categories

$$\begin{aligned} \gamma (P,k,y) :{{\,\textrm{Nil}\,}}(R_{\rho |_P}[P],\psi _{(P,k,y)}) \rightarrow {{\,\textrm{Nil}\,}}(R_{\rho }[F],\psi ) \end{aligned}$$

by sending an object in ${{\,\textrm{Nil}\,}}(R[P],\psi |_P)$ given by a nilpotent *R*[*P*]-endomorphism $\varphi :(c_y \circ \alpha ^k)_* Q = RP \otimes _{c_y \circ \alpha ^k} Q \rightarrow Q$ for a finitely generated projective *R*[*P*]-module *Q* to the object in ${{\,\textrm{Nil}\,}}(R[F],\alpha ^k)$ given by the nilpotent *R*[*F*]-endomorphism $(\alpha ^k)_* \bigl (R[F] \otimes _{R[P]} Q\bigr ) = RF \otimes _{\alpha ^k} \bigl (R[F] \otimes _{R[P]} Q\bigr ) \rightarrow R[F] \otimes _{R[P]} Q$ sending $f_0 \otimes (f_1 \otimes q)$ to $(\alpha ^{-k}(f_0 ) f_1t^{-k}y^{-1}) \otimes \varphi (1 \otimes q)$. It induces for every $n \in \mathbb {Z}$ a homomorphism

$$\begin{aligned} \gamma (P,k,y)_m :N\!K_n(R[P],c_y \circ \alpha ^k) \rightarrow N\!K_n(R[F],\alpha ^k). \end{aligned}$$

Let

$$\begin{aligned} (V_k)_n :N\!K_n(R[F],\alpha ^k) \rightarrow N\!K_n(R[F],\alpha ) \end{aligned}$$

be the homomorphism induced by the Verschiebungs operator $V_k$, see (3.2).

#### Theorem 1.13

The homomorphism

$$\begin{aligned} \bigoplus _{(P,k,y) \in T_p}\bigl ((V_K)_n \circ \gamma (P,k,y)_n\bigr )_{(p)} :\bigoplus _{(P,k,y) \in T_p} N\!K_n(R[P],c_y \circ \alpha ^k)_{(p)} \rightarrow N\!K_n(R[F],\alpha )_{(p)} \end{aligned}$$

is surjective for every $n \in \mathbb {Z}$, where the subscript (*p*) stands for localization at the prime *p*.

The untwisted version of Theorem 1.13, i.e., $\alpha = {{\,\textrm{id}\,}}_F$ and trivial $\mu$, appears already in [21, Theorem A].

Note that this does not mean that the Nil-groups are computable after localizing at *p* by *p*-subgroups groups, since the maps $\gamma (P,k,y)_n$ are not given just by induction with the inclusion $P \rightarrow F$. One can check by inspecting Lemmas 7.6 and 8.11 that the Nil-groups are computable by *p*-elementary groups.

#### Corollary 1.14

Let *R* be a regular ring. Then $\mathbb {Z}[\mathcal {P}(F,R)^{-1}] \otimes _{\mathbb {Z}} N\!K_n(R[F],\alpha )$ vanishes for $\mathcal {P}(F,R)$ defined in Notation 1.1.

We mention that the second Nil-group of $F_2[\mathbb {Z}/2]$ is non-trivial, see [40]. So one needs to invert certain primes in Corollary 1.14.

On the other hand, given a prime *p*, we get $N\!K_n(\mathbb {Z}[\mathbb {Z}/p])=0$ for $n \le 1$, see [11, Theorem 10.6 on page 695], [12, 25, Theorem 6.21]. So Theorem 1.13 implies that for a finite group *G*, for which $p^2$ does not divide the order of *G*, we have $N\!K_n(\mathbb {Z}G)_{(p)} = 0$ for $n \le 1$. As an application we get a new proof of the result of Harmon [22] that $N\!K _n(\mathbb {Z}G) = 0$ for $n \le 1$ if the order of *G* is square-free.

#### Remark 1.15

We mention without giving the details that the proof appearing in [25, Theorem 6.21] can be generalized to the twisted setting showing that Harmon’s result extends to twisted group rings and that in Theorem 1.13 the terms $N\!K_n(R[P],c_y \circ \alpha ^k)_{(p)}$ vanish if $|P| \le p$, $R= \mathbb {Z}$, and $n \le 1$ hold. This implies that for a group *G* for which the order of any finite subgroup is squarefree the relative assembly map

$$\begin{aligned} H_n^G(E_{{{{\mathcal {F}}}\textrm{in}}}(G);\textbf{K}_\mathbb {Z}) \rightarrow H_n^G(E_{{{{\mathcal {V}}}\textrm{cyc}}}(G);\textbf{K}_\mathbb {Z}) \end{aligned}$$

is an isomorphism for every $n \in \mathbb {Z}$ with $n \le 1$. If *G* satisfies the Full Farrell–Jones Conjecture and the order of any finite subgroup is squarefree, then the assembly map

$$\begin{aligned} H_n^G(E_{{{{\mathcal {F}}}\textrm{in}}}(G);\textbf{K}_\mathbb {Z}) \rightarrow H_n^G(G/G;\textbf{K}_\mathbb {Z}) = K_n(\mathbb {Z}[G]) \end{aligned}$$

is an isomorphism for every $n \in \mathbb {Z}$ with $n \le 1$. Examples for such *G* are given by extensions $1 \rightarrow \mathbb {Z}^n \rightarrow G \rightarrow \mathbb {Z}/m \rightarrow 1$ for a squarefree natural number *m*.

#### Remark 1.16

(*K-theory of stable *$\infty$*-categories*) One may ask whether the results of this paper can be extended from the *K*-theory of additive categories to the *K*-theory of stable $\infty$-categories. For the extension of the statement and proofs in some cases of the Full Farrell–Jones Conjecture, we refer to Bunke–Kasprowski–Winges [15]. Dominik Kirstein and Christian Kremer are working on a twisted Bass–Heller–Swan decomposition in this setting generalizing [29, 33]. Efimov has announced that the Nil-terms are modules over TR on the spectrum level, which would yield a module structure of Nil-groups over the ring of big Witt vectors also for stable $\infty$-categories. However, algebraic *K*-theory for additive categories can be viewed as a Mackey functor over the Green functor given by the Swan ring, see Sect. 5.4. This is very unlikely to be the case for the algebraic *K*-theory of stable $\infty$-categories, where an *A*-theoretic version of the Swan group is needed, see [39]. Therefore the induction theorems, which we use here for instance to prove Theorem 1.13, are not available in the setting of stable $\infty$-categories. It is completely unclear in the setting of stable $\infty$-categories whether Theorems 1.2 or 1.13 are still true and how one can formulate the statement of Theorem 1.12.

## Basics about (additive) $\Lambda$-categories

Consider a commutative ring $\Lambda$ and a group *G*. A $\Lambda$-*category* is a small category $\mathcal {A}$ enriched over the category of $\Lambda$-modules, i.e., for every two objects *A* and $A'$ in $\mathcal {A}$ the set of morphisms  has the structure of a $\Lambda$-module such that composition is a $\Lambda$-bilinear map. A *G*-$\Lambda$*-category* is a $\Lambda$-category, which comes with a *G*-action by automorphisms of $\Lambda$-categories.

If a $\Lambda$-category comes with an appropriate notion of a finite direct sums, it is called an *additive* $\Lambda$*-category*. An *additive* *G*-$\Lambda$*-category* is an additive $\Lambda$-category, which comes with a *G*-action by automorphisms of additive $\Lambda$-categories. If $\Lambda$ is $\mathbb {Z}$, we often omit $\Lambda$ and talk just about an additive category or additive *G*-category.

One can associate to a $\Lambda$-category category $\mathcal {A}$ an additive $\Lambda$-category $\mathcal {A}_{\oplus }$ as follows. Objects in $\mathcal {A}_{\oplus }$ are pairs  consisting of a finite set *S* and a map  and a morphism  is given by a collection  of morphisms in $\mathcal {A}$ for $s \in S$ and $s' \in S'$. The direct sum  is given by $.$

Note that a $\Lambda$-category and an additive $\Lambda$-category respectively is in particular a $\mathbb {Z}$-category and an additive category respectively thanks to the canonical ring homomorphism $\mathbb {Z}\rightarrow \Lambda$.

One can assign to an additive category $\mathcal {A}$ its non-connective *K*-theory spectrum $\textbf{K}(\mathcal {A})$. We denote $K_n(\mathcal {A}) = \pi _n(\textbf{K}(\mathcal {A}))$ for $n \in \mathbb {Z}$.

Given a ring *R*, define $\underline{R}$ to be the $\mathbb {Z}$-category, which has precisely one object $*_R$ and whose $\mathbb {Z}$-module of endomorphisms is *R*. Composition is given by the multiplication in *R*.

For a ring *R* let $K_n(R)$ for $n \in \mathbb {Z}$ be its *n*-algebraic *K*-group, which can be defined for instance as the (non-connective) *K*-theory of the exact category of finitely generated projective *R*-module. It can be identified with $K_n(\underline{R}_{\oplus })$. Note that we do not have to pass to the idempotent completion of $\underline{R}_{\oplus }$, as we are working with non-connective *K*-theory.

All these classical notions are summarized with references to the relevant papers in [4, Section 2 and 3].

We fix some conventions concerning matrices of morphisms. For an object *A* in $\mathcal {A}$ we denote by $A^m$ the direct sum $\bigoplus _{i=1}^m A$. For two finite direct sums $\bigoplus _{i= 1}^m A_i$ and $\bigoplus _{j= 1}^n B_j$ a morphism $U :\bigoplus _{i= 1}^m A_i \rightarrow \bigoplus _{j= 1}^n B_j$ is the same as a (*m*, *n*)-matrix

$$\begin{aligned} U = \begin{pmatrix} u_{1,1} &  u_{2,1} &  u_{3,1} &  \cdots &  u_{m-2,1} &  u_{m-1,1} &  u_{m,1} \\ u_{1,2} &  u_{2,2} &  u_{3,2} &  \cdots &  u_{m-2,2} &  u_{m-1,2} &  u_{m,1} \\ u_{1,3} &  u_{2,3} &  u_{3,3} &  \cdots &  u_{m-2,3} &  u_{m-1,3} &  u_{m,3} \\ \vdots &  \vdots &  \vdots &  \ddots & \vdots &  \vdots &  \vdots \\ u_{1,n-1} &  u_{2,n-1} &  u_{3,n-1} &  \cdots &  u_{m-2,n-1} &  u_{m-1,n-1} &  u_{m,n-1} \\ u_{1,n} &  u_{2,n} &  u_{3,n} &  \cdots &  u_{m-2,n} &  u_{m-1,n} &  u_{m,n} \end{pmatrix} \end{aligned}$$

of morphisms $u_{i,j} :A_i \rightarrow B_j$. One may think of an element in $\bigoplus _{i= 1}^m A_i$ as a (1, *m*)-matrix

$$\begin{aligned} x:= \begin{pmatrix} x_1 \\ x_2 \\ \vdots \\ x_m \end{pmatrix} \end{aligned}$$

and thinks of the image of this element under *U* as the (1, *n*)-matrix

$$\begin{aligned}U(x) = \begin{pmatrix} \sum _{l = 1}^m u_{l,1}(x_l) \\ \sum _{l = 1}^m u_{l,2}(x_l) \\ \vdots \\ \sum _{l = 1}^m u_{l,n}(x_l) \end{pmatrix}. \end{aligned}$$

Note that *m* is the number of columns and *n* is the number of rows with these conventions. If $V :\bigoplus _{j= 1}^n B_j \rightarrow \bigoplus _{k= 1}^o C_k$ is another morphism given by the (*n*, *o*)-matrix *V*, then the composite $V \circ U :\bigoplus _{i= 1}^m A_i \rightarrow \bigoplus _{k= 1}^o C_k$ is given by the (*m*, *o*) matrix *W* whose (*i*, *k*)-entry is

$$\begin{aligned} w_{i,k} = \sum _{j = 1}^n v_{j,k} \circ u_{i,j}. \end{aligned}$$

So one could think of *W* as a kind of product of matrices $V \cdot U$.

## Frobenius and Verschiebungs operators

Let $\mathcal {A}$ be an additive category and $\Phi$ be an automorphism of $\mathcal {A}$.

### Definition 3.1

(*Nilpotent morphisms and Nil-categories*)

(i) A morphism $\varphi :\Phi (A)\rightarrow A$ of $\mathcal {A}$ is called $\Phi$-*nilpotent*, if for some $n \ge 1$ the *n*-fold composite
  $$\begin{aligned} \varphi ^{(n)}:=\varphi \circ \Phi (\varphi ) \circ \cdots \circ \Phi ^{n-1}(\varphi ) :\Phi ^n(A)\rightarrow A. \end{aligned}$$
  is trivial;
(ii) The category ${{\,\textrm{Nil}\,}}(\mathcal {A}, \Phi )$ has as objects pairs $(A, \varphi )$, where $\varphi :\Phi (A)\rightarrow A$ is a $\Phi$-nilpotent morphism in $\mathcal {A}$. A morphism from $(A, \varphi )$ to $(A', \varphi ')$ is a morphism $u:A\rightarrow A'$ in $\mathcal {A}$ such that the diagram  is commutative.

The category ${{\,\textrm{Nil}\,}}(\mathcal {A}, \Phi )$ inherits the structure of an exact category from $\mathcal {A}$, a sequence in ${{\,\textrm{Nil}\,}}(\mathcal {A},\Phi )$ is declared to be exact if the underlying sequence in $\mathcal {A}$ is (split) exact.

Next we define for $k \in \{1,2, \ldots \}$ the *Verschiebung operator* $V_k$ and the *Frobenius operator* $F_k$

3.2 $$\begin{aligned} V_k :{{\,\textrm{Nil}\,}}(\mathcal {A}, \Phi ^k)\rightarrow &   {{\,\textrm{Nil}\,}}(\mathcal {A}, \Phi ); \end{aligned}$$

3.3 $$\begin{aligned} F_k :{{\,\textrm{Nil}\,}}(\mathcal {A}, \Phi )\rightarrow &   {{\,\textrm{Nil}\,}}(\mathcal {A}, \Phi ^k). \end{aligned}$$

Given an object $(A, \varphi )$ in ${{\,\textrm{Nil}\,}}(\mathcal {A}, \Phi ^k)$, define $V_k(A, \varphi )$ to be the object in ${{\,\textrm{Nil}\,}}(\mathcal {A}, \Phi )$ that is given by the object $\bigoplus _{i =0}^{k-1} \Phi ^i(A)$ in $\mathcal {A}$ together with the $\Phi$-nilpotent morphism

$$\begin{aligned} \begin{pmatrix} 0 &  0 &  0 &  0 &  0 &  \cdots &  0 &  0 &  \varphi \\ {{\,\textrm{id}\,}}_{\Phi (A)} &  0 &  0 &  0 &  0 &  \cdots &  0 &  0 &  0 \\ 0 &  {{\,\textrm{id}\,}}_{\Phi ^2(A)} &  0 &  0 &  0 & \cdots &  0 &  0 &  0 \\ 0 &  0 & {{\,\textrm{id}\,}}_{\Phi ^3(A)} &  0 &  0 &  \cdots &  0 &  0 &  0 \\ \vdots &  \vdots &  \vdots & \vdots &  \vdots &  \vdots &  \vdots &  \vdots &  \vdots \\ 0 &  0 &  0 &  0 &  0 &  \cdots &  {{\,\textrm{id}\,}}_{\Phi ^{k-2}(A)} &  0 &  0 \\ 0 &  0 &  0 &  0 &  0 &  \cdots &  0 &  {{\,\textrm{id}\,}}_{\Phi ^{k-1}(A)} &  0 \end{pmatrix} :\\ \Phi \left( \bigoplus _{i =0}^{k-1} \Phi ^i(A)\right) =\bigoplus _{i =1}^k \Phi ^i(A) \rightarrow \bigoplus _{i =0}^{k-1} \Phi ^i(A). \end{aligned}$$

A morphisms $u :(A, \varphi ) \rightarrow (A', \varphi ')$ in ${{\,\textrm{Nil}\,}}(\mathcal {A}, \Phi ^k)$ given by a morphism $u :A \rightarrow A'$ in $\mathcal {A}$ is sent to the morphism $V_k(A,\varphi ) \rightarrow V_k(A',\varphi ')$ in ${{\,\textrm{Nil}\,}}(\mathcal {A}, \Phi )$ given by the morphism $\bigoplus _{i =0}^{k-1} \Phi ^i(u) :\bigoplus _{i =0}^{k-1} \Phi ^i(A) \rightarrow \bigoplus _{i =0}^{k-1} \Phi ^i(A')$.

Given an object $(A, \varphi )$ in ${{\,\textrm{Nil}\,}}(\mathcal {A}, \Phi )$, define $F_k(A, \varphi )$ to be $(A, \varphi ^{(k)})$, see Definition 3.1. A morphism $u :(A,\varphi ) \rightarrow (A',\varphi ')$ in ${{\,\textrm{Nil}\,}}(\mathcal {A}, \Phi )$ given by a morphism $u :A \rightarrow A'$ in $\mathcal {A}$ is sent to the morphism $u :(A,\varphi ^{(k)}) \rightarrow (A',\varphi '^{(k)})$ in ${{\,\textrm{Nil}\,}}(\mathcal {A}, \Phi ^k)$ given by *u* again.

The elementary proof of the next lemma is left to the reader.

### Lemma 3.4

The composite

$$\begin{aligned} F_k \circ V_k :{{\,\textrm{Nil}\,}}(\mathcal {A}, \Phi ^k) \rightarrow {{\,\textrm{Nil}\,}}(\mathcal {A}, \Phi ^k) \end{aligned}$$

sends an object $(A,\varphi )$ to the object given by

$$\begin{aligned} \bigoplus _{i = 0}^{k-1}\Phi ^i(\varphi ):\Phi ^k\left( \bigoplus _{i= 0}^{k-1} \Phi ^i(A)\right) = \bigoplus _{i= 0}^{k-1} \Phi ^{i+k}(A) \rightarrow \bigoplus _{i= 0}^{k-1} \Phi ^i(A). \end{aligned}$$

It sends a morphism $u :(A,\varphi ) \rightarrow (A',\varphi ')$ in ${{\,\textrm{Nil}\,}}(\mathcal {A}, \Phi ^k)$ given by a morphism $u :A \rightarrow A'$ in $\mathcal {A}$ to the morphism $F_k \circ V_k(A,\varphi ) \rightarrow F_k \circ V_k(A',\varphi ')$ in ${{\,\textrm{Nil}\,}}(\mathcal {A}, \Phi ^k)$ given by the morphism $\bigoplus _{i= 0}^{k-1} \Phi ^i(u) :\bigoplus _{i= 0}^{k-1} \Phi ^i(A) \rightarrow \bigoplus _{i= 0}^{k-1} \Phi ^i(A')$ in $\mathcal {A}$.

## Frobenius and Verschiebungs operators and induction and restriction

Let $\mathcal {A}$ be an additive category with an action $\rho :G \rightarrow {{\,\textrm{aut}\,}}(\mathcal {A})$ of the (discrete) group *G* by automorphisms of additive categories. Then we obtain a new additive category

4.1 $$\begin{aligned} \mathcal {A}_{\rho }[G] \end{aligned}$$

as follows. The set of objects of $\mathcal {A}_{\rho }[G]$ is the set of objects of $\mathcal {A}$. A morphism $f :A \rightarrow A'$ in $\mathcal {A}_{\rho }[G]$ is a finite formal $\sum _{g \in G} (f_g :gA \rightarrow A') \cdot g$, where $f_g :gA \rightarrow A'$ is a morphism in $\mathcal {A}$ from *gA* to $A'$ and finite means that for only finitely many elements *g* in *G* the morphism $f_g$ is different from the zero-homomorphism. If $f' :A' \rightarrow A''$ is a morphism in $\mathcal {A}_{\rho }[G]$ given by the finite formal sum $\sum _{g' \in G} (f'_{g'} :g'A' \rightarrow A'') \cdot g'$, then define their composite $f' \circ f :A \rightarrow A''$ by the finite formal sum

$$\begin{aligned} f' \circ f = \sum _{g'' \in G} \; \sum _{\begin{array}{c} g,g' \in G,\\ g'' = g'g \end{array}} \; (f_{g'} \circ g'f_{g} :g''A = g'gA \rightarrow A'') \cdot g''. \end{aligned}$$

If *R* is a unital ring coming with a *G*-action $\rho _R :G \rightarrow {{\,\textrm{aut}\,}}(R)$ and we take $\mathcal {A}$ to be the category of finitely generated free *R*-modules with the obvious *G*-action $\rho :G \rightarrow {{\,\textrm{aut}\,}}(\mathcal {A})$ coming from $\rho _R$ by induction, then $\mathcal {A}_{\rho }[G]$ is equivalent to the additive category of finitely generated free modules over the twisted group ring $R_{\rho }[G]$.

If $\Phi :\mathcal {A}\xrightarrow {\cong } \mathcal {A}$ is an automorphism of an additive category $\mathcal {A}$, then we define

4.2 $$\begin{aligned} \mathcal {A}_{\Phi }[\mathbb {Z}] = \mathcal {A}_{\rho _{\Phi }}[\mathbb {Z}] \end{aligned}$$

for the $\mathbb {Z}$-action $\rho _{\Phi } :\mathbb {Z}\rightarrow {{\,\textrm{aut}\,}}(\mathcal {A}), \; n \mapsto \Phi ^n$.

Let $i_k :\mathbb {Z}\rightarrow \mathbb {Z}$ be the group homomorphism given by multiplication with *k*. Next we define functors of additive categories

4.3 $$\begin{aligned} (i_k)_* :\mathcal {A}_{\Phi ^k}[\mathbb {Z}]\rightarrow &   \mathcal {A}_{\Phi }[\mathbb {Z}]; \end{aligned}$$

4.4 $$\begin{aligned} i_k^* :\mathcal {A}_{\Phi }[\mathbb {Z}]\rightarrow &   \mathcal {A}_{\Phi ^k}[\mathbb {Z}]. \end{aligned}$$

The functor $(i_k)_*$ sends an object *A* in $\mathcal {A}_{\Phi ^k}[\mathbb {Z}]$, which is given by an object *A* in $\mathcal {A}$, to the object in $\mathcal {A}_{\Phi }[\mathbb {Z}]$ given by *A* again. Consider a morphism

$$\begin{aligned} f = \sum _{l \in \mathbb {Z}} \bigl (f_l :\Phi ^{kl}(A) \rightarrow A'\bigr ) \cdot t^l :A \rightarrow A' \end{aligned}$$

in $\mathcal {A}_{\Phi ^k}[\mathbb {Z}]$. It is sent by $( i_k)_*$ to the morphism

$$\begin{aligned} (i_k)_*(f):= \sum _{l \in \mathbb {Z}} \bigl (f_l :\Phi ^{kl}(A) \rightarrow A'\bigr ) \cdot t^{lk} :A \rightarrow A' \end{aligned}$$

in $$\mathcal {A}_{\Phi }[\mathbb {Z}]$$.

The functor $i_k^*$ sends a object *A* in $\mathcal {A}_{\Phi }[\mathbb {Z}]$, which is given by an object *A* in $\mathcal {A}$, to the object $i_k^*(A)$ in $\mathcal {A}_{\Phi ^k}[\mathbb {Z}]$ given by the object $\bigoplus _{i = 0}^{k-1} \Phi ^i(A)$ in $\mathcal {A}$. Consider a morphism $f = \sum _{l \in \mathbb {Z}} \bigl (f_l :\Phi ^{l}(A) \rightarrow A'\bigr ) \cdot t^l :A \rightarrow A'$ in $\mathcal {A}_{\Phi }[\mathbb {Z}]$. It is sent by $i_k^*$ to the morphism

$$\begin{aligned} i_k^*(f) :\bigoplus _{i = 0}^{k-1} \Phi ^i(A) \rightarrow \bigoplus _{j = 0}^{k-1} \Phi ^j(A') \end{aligned}$$

in $\mathcal {A}_{\Phi }[\mathbb {Z}]$ defined as follows. By additivity we have only to specify $i_k^*(f_l \cdot t^l)$. For this purpose we have to define for every $i,j \in \{0,1,\ldots , (k-1)\}$ a morphisms $i_k^*(f_l \cdot t^l)_{i,j} :\Phi ^i(A) \rightarrow \Phi ^j(A')$ in $\mathcal {A}_{\Phi ^k}[\mathbb {Z}]$. It is given by $\bigl (\Phi ^j(f_l) :\Phi ^{i+mk}(A) \rightarrow \Phi ^j(A')\bigr ) \cdot t^m$ if there exists an integer *m* with $i+mk +l =j$, and by zero otherwise.

If $\mathcal {A}$ is given by $\underline{R}$ for a ring *R* coming with a ring automorphism $\Phi :R \xrightarrow {\cong } R$, $(i_k)_*$ and $i_k^*$ corresponds to induction and restriction with respect to the change of ring homomorphism of twisted group rings $R_{\Phi ^k}[\mathbb {Z}] \rightarrow R_{\Phi }[\mathbb {Z}]$ associated to $i_k$.

In the sequel we use the notation of [29]. We get by taking homotopy groups from [29, Theorem 0.1] for $n \in \mathbb {Z}$ an isomorphism

4.5 $$\begin{aligned} a_n \oplus c_n^+ \oplus c_n^- :\pi _n(\textbf{T}_{\textbf{K}(\Phi ^{-1})}) \oplus \overline{K}_{n-1}({{\,\textrm{Nil}\,}}(\mathcal {A},\Phi )) \oplus \overline{K}_{n-1}({{\,\textrm{Nil}\,}}(\mathcal {A},\Phi )) \xrightarrow {\cong } K_n(\mathcal {A}_{\Phi }[\mathbb {Z}]). \nonumber \\ \end{aligned}$$

Here $\textbf{T}_{\textbf{K}(\Phi ^{-1})}$ is the mapping torus of the map induced on non-connective *K*-theory spectra $\textbf{K}(\Phi ^{-1}) :\textbf{K}(\mathcal {A}) \rightarrow \textbf{K}(\mathcal {A})$. There is a long exact Wang sequence

$$\begin{aligned}  &   \cdots \xrightarrow {\partial _{n+1}} K_n(\mathcal {A}) \xrightarrow {K_n(\Phi ) - {{\,\textrm{id}\,}}} K_n(\mathcal {A}) \xrightarrow {\pi _n(\textbf{j})} \pi _n(\textbf{T}_{\textbf{K}(\Phi ^{-1})})\\  &   \quad \xrightarrow {\partial _n} K_{n-1}(\mathcal {A}) \xrightarrow {K_{n-1}(\Phi ) - {{\,\textrm{id}\,}}} K_{n-1}(\mathcal {A}) \xrightarrow {\pi _{n-1}(\textbf{j})} \pi _n(\textbf{T}_{\textbf{K}(\Phi ^{-1})}) \xrightarrow {\partial _{n-1}} \cdots \end{aligned}$$

where $\textbf{j}:\textbf{K}(\mathcal {A}) \rightarrow \textbf{T}_{\textbf{K}(\Phi ^{-1})}$ is the inclusion. If $\Phi = {{\,\textrm{id}\,}}_{\mathcal {A}}$, this boils down to an isomorphism

$$\begin{aligned} \pi _n(\textbf{T}_{\textbf{K}(\Phi ^{-1})}) \cong K_n(\mathcal {A}) \oplus K_{n-1}(\mathcal {A}). \end{aligned}$$

We define $K_{n}({{\,\textrm{Nil}\,}}(A,\Phi ^k)) = \pi _n(\textbf{K}_{{{\,\textrm{Nil}\,}}}^{\infty }(\mathcal {A},\Phi ^k))$ for $n \in \mathbb {Z}$, where the non-connective *K*-theory spectrum $\textbf{K}_{{{\,\textrm{Nil}\,}}}^{\infty }(\mathcal {A},\Phi )$ is constructed in [27, Remark 6.3 and Lemma 6.5]. It is likely but we have no detailed proof that the group $K_n({{\,\textrm{Nil}\,}}(\mathcal {A},\Phi ))$ can be identified for $n \le -1$ with the *K*-groups associated to the exact category ${{\,\textrm{Nil}\,}}(\mathcal {A},\Phi )$ in the sense of Schlichting [34] see [27, Remark 6.11]. Fortunately, we do not need this identification for our purposes. There is the inclusion functor $I :\mathcal {A}\rightarrow {{\,\textrm{Nil}\,}}(\mathcal {A},\Phi )$ that sends an object *A* to the object (*A*, 0) and the projection functor $P :{{\,\textrm{Nil}\,}}(\mathcal {A},\Phi ) \rightarrow \mathcal {A}$ that sends an object $(A,\varphi )$ to *A*. Obviously $P \circ I = {{\,\textrm{id}\,}}_{\mathcal {A}}$.

### Notation 4.6

We define $\overline{K}_n({{\,\textrm{Nil}\,}}(A,\Phi ))$ to be the cokernel of the split injective homomorphism $K_n(I) :K_n(\mathcal {A}) \rightarrow K_n({{\,\textrm{Nil}\,}}(A,\Phi ))$ for $n \in \mathbb {Z}$.

Let $T_{\textbf{K}(\Phi ^{-1}),k}$ be the *k*-fold mapping torus of $\textbf{K}(\Phi ) :\textbf{K}(\mathcal {A}) \rightarrow \textbf{K}(\mathcal {A})$, which is a $\mathbb {Z}/k$-spectrum. There is a *k*-fold covering $\textbf{p}_k :T_{\textbf{K}(\Phi ^{-1}),k} \rightarrow T_{\textbf{K}(\Phi ^{-1})}$ and a homotopy equivalence $\textbf{f}:T_{\textbf{K}(\Phi ^{-1}),k} \rightarrow \textbf{T}_{\textbf{K}(\Phi ^{-k})}$. These correspond to the following construction on the level of spaces for a map $\Phi :X \rightarrow X$. Namely, $p_k :T_{\Phi ,k} \rightarrow T_{\Phi }$ is the *k*-sheeted covering obtained by the pull back of the *k*-sheeted covering $S^1 \rightarrow S^1$ sending *z* to $z^k$ with the canonical map $T_{\Phi } \rightarrow S^1$. Explicitly $T_{\Phi ,k}$ is obtained from $\coprod _{i = 1}^{k} X \times [i-1,i]$ by identifying $(x,i) \in X \times [i-1,i]$ with $(\Phi (x),i)$ in $X \times [i,i+1]$ for $i = 1,2 \ldots k-1$ and $(x,k) \in X \times [k-1,k]$ with $(\Phi (x),0)$ in $X \times [0,1]$. Obviously $T_{\Phi ,1} = T_{\Phi }$. The map $p_k :T_{\Phi ,k} \rightarrow T_{\Phi }$ sends the class of $(x, j) \in X \times [i-1,i]$ to the class of $(x,j-i-1) \in X \times [0,1]$ for $i = 1,2, \ldots , k$. The homotopy equivalence $f :T_{\Phi ,k} \rightarrow T_{\Phi ^k}$ sends the class of $(x, j) \in X \times [i-1,i]$ to the class of $(\Phi ^{k-i}(x),\frac{j}{k}) \in X \times [0,1]$ for $i = 1,2, \ldots , k$. On homotopy groups we obtain an isomorphism

4.7 $$\begin{aligned} \pi _n(\textbf{f}) :\pi _n(\textbf{T}_{\Phi ^{-1},k}) \xrightarrow {\cong } \pi _n(T_{\Phi ^{-k}}) \end{aligned}$$

and a homomorphism induced by $\textbf{p}_k$

4.8 $$\begin{aligned} \pi _n(\textbf{p}_k) :\pi _n(\textbf{T}_{\Phi ^{-1},k}) \xrightarrow {\cong } \pi _n(T_{\Phi ^{-1}}). \end{aligned}$$

Since $\textbf{p}_k$ is a *k*-sheeted covering, there is a transfer homomorphism

4.9 $$\begin{aligned} {\text {trf}}_n(\textbf{p}_k) :\pi _n(T_{\Phi ^{-1}}) \rightarrow \pi _n(\textbf{T}_{\Phi ^{-1},k}). \end{aligned}$$

Note that the Frobenius and the Verschiebungs operator are functors of exact categories and hence induces homomorphism

4.10 $$\begin{aligned} K_n(V_k) :K_n({{\,\textrm{Nil}\,}}(\mathcal {A},\Phi ^k) )\rightarrow &   K_n({{\,\textrm{Nil}\,}}(\mathcal {A},\Phi )); \end{aligned}$$

4.11 $$\begin{aligned} K_n(F_k) :K_n({{\,\textrm{Nil}\,}}(\mathcal {A},\Phi ))\rightarrow &   K_n({{\,\textrm{Nil}\,}}(\mathcal {A},\Phi ^k)). \end{aligned}$$

Since $K_n(F_k) \circ K_n(I) = K_n(I)$ and $K_n(V_k) \circ K_n(I) = K_n(I)$ holds, they induce homomorphisms

4.12 $$\begin{aligned} \overline{K}_n(V_k) :\overline{K}_n({{\,\textrm{Nil}\,}}(\mathcal {A},\Phi ^k) )\rightarrow &   \overline{K}_n({{\,\textrm{Nil}\,}}(\mathcal {A},\Phi )); \end{aligned}$$

4.13 $$\begin{aligned} \overline{K}_n(F_k) :\overline{K}_n({{\,\textrm{Nil}\,}}(\mathcal {A},\Phi ))\rightarrow &   \overline{K}_n({{\,\textrm{Nil}\,}}(\mathcal {A},\Phi ^k)). \end{aligned}$$

The functors $(i_k)_*$ and $i_k^*$ are functors of additive categories and induce homomorphisms

4.14 $$\begin{aligned} K_n((i_k)_*) :K_n(\mathcal {A}_{\Phi ^k}[\mathbb {Z}])\rightarrow &   K_n(\mathcal {A}_{\Phi }[\mathbb {Z}]); \end{aligned}$$

4.15 $$\begin{aligned} K_n(i_k^*) :K_n(\mathcal {A}_{\Phi }[\mathbb {Z}])\rightarrow &   K_n(\mathcal {A}_{\Phi ^k}[\mathbb {Z}]). \end{aligned}$$

The main result of this section is

### Theorem 4.16

Let $k \ge 1$ be a natural number and $\Phi :\mathcal {A}\xrightarrow {\cong } \mathcal {A}$ be an automorphism of an additive category $\mathcal {A}$. Then:

(i) The following diagram commutes for $n \in \mathbb {Z}$ where the upper horizontal isomorphisms is the one defined in (4.5) for $\Phi ^k$, the lower horizontal isomorphisms is the one defined in (4.5) for $\Phi$, and the vertical arrows have been defined in (4.7), (4.8), (4.12), and (4.14);
(ii) The following diagram commutes for $n \in \mathbb {Z}$ where the upper isomorphisms is the one defined in (4.5) for $\Phi$, the lower isomorphisms is the one defined in (4.5) for $\Phi ^k$, and the horizontal arrows have been defined in (4.7), (4.9), (4.13), and (4.15).

### Proof

(i) We give only an outline of the proof and leave some details to the reader. In the sequel we use the notation of [29].

We obtain from [29, Theorem 0.1 (i)] for $n \in \mathbb {Z}$ an isomorphism

4.17 $$\begin{aligned} a_n \oplus b_n^+ \oplus b_n^- :\pi _n(\textbf{T}_{\textbf{K}(\Phi ^{-1})}) \oplus N\!K_n(\mathcal {A}_{\Phi }[t]) \oplus N\!K_n(\mathcal {A}_{\Phi }[t^{-1}]) \xrightarrow {\cong } K_n(\mathcal {A}_{\Phi }[\mathbb {Z}]), \nonumber \\ \end{aligned}$$

where $N\!K_n(\mathcal {A}_{\Phi }[t^{\pm }])$ is the kernel of the map $K_n(\mathcal {A}_{\Phi }[t^{\pm }]) \rightarrow K_n(\mathcal {A})$ coming from the functor $\mathcal {A}_{\Phi }[t^{\pm }] \rightarrow \mathcal {A}$ given taking the coefficient of $t^0$. One easily checks that the functors $(i_k)_*$ and $i_k^*$ of (4.3) and (4.4) induce homomorphisms

4.18 $$\begin{aligned} N\!K_n((i_k)_*)_{\pm } :N\!K_n(\mathcal {A}_{\Phi ^k}[t^{\pm 1}])\rightarrow &   N\!K_n(\mathcal {A}_{\Phi }[t^{\pm 1}]); \end{aligned}$$

4.19 $$\begin{aligned} N\!K_n(i_k^*)_{\pm } :N\!K_n(\mathcal {A}_{\Phi }[t^{\pm 1}])\rightarrow &   N\!K_n(\mathcal {A}_{\Phi ^k}[t^{\pm 1}]). \end{aligned}$$

Then following diagram commutes for $n \in \mathbb {Z}$where the upper horizontal arrow is the isomorphism (4.17) for $\Phi ^k$, the lower horizontal arrow is the isomorphism (4.17) for $\Phi$ and the vertical homomorphism have been defined in (4.7), (4.8), (4.14) and (4.18). The proof of commutativity for the terms $N\!K_n(\mathcal {A}_{\Phi ^k}[t])$ and $N\!K_n(\mathcal {A}_{\Phi ^k}[t^{-1}])$ is obvious, the one for the term $\pi _n(\textbf{T}_{\textbf{K}(\Phi ^{-k})})$ is left to the reader.

We obtain from [29, Theorem 0.1 (ii)] for $n \in \mathbb {Z}$ an isomorphism

4.20 $$\begin{aligned} \alpha (\Phi ,\pm )_n :K_{n-1}(\mathcal {A}) \oplus N\!K_n(\mathcal {A}_{\Phi }[t^{\pm }])&\xrightarrow {\cong }&K_{n-1}({{\,\textrm{Nil}\,}}(\mathcal {A},\Phi )). \end{aligned}$$

and hence an isomorphism

4.21 $$\begin{aligned} \widetilde{\alpha }(\Phi ,\pm )_n :N\!K_n(\mathcal {A}_{\Phi }[t^{\pm }])&\xrightarrow {\cong }&\overline{K}_{n-1}({{\,\textrm{Nil}\,}}(\mathcal {A},\Phi )). \end{aligned}$$

Therefore it remains to show that the diagram4.22commutes for $n \in \mathbb {Z}$. Fix a natural number *m*. Then the diagram (4.22) is a retract of the corresponding diagramwhere we have replaced $\mathcal {A}$ by $\mathcal {A}[\mathbb {Z}^m]$ and $\Phi$ by $\Phi [\mathbb {Z}^m]$, see [27, Lemma 2.2, Theorem 6.2, Remark 6.3]. Hence we can assume without loss of generality that $n \ge 1$ when proving the commutativity of (4.22), since our result shall be true for any additive category $\mathcal {A}$. So we can use the connective *K*-theory spectrum when dealing with the commutativity of (4.22). By inspecting [29] one sees that this boils down to show that the following diagram commutes for $n \ge 1$,4.23where the upper and the lower homomorphism are induced by the functors4.244.25introduced in [29, Section 8], the left horizontal arrow has been introduced in (4.10) and the right vertical arrow is induced by the functor $(i_k)_*$ of (4.3). Recall that  is the category of bounded chain complexes over $\mathcal {A}_\Phi [t^{-1}]$, which are contractible as chain complexes over $\mathcal {A}_\Phi [t,t^{-1}]$, and that the lower horizontal arrow sends an object $(A,\varphi )$ in ${{\,\textrm{Nil}\,}}(\mathcal {A},\Phi )$ to the $\mathcal {A}_{\Phi }[t^{-1}]$-chain complex concentrated in dimensions 0 and 1, whose first differential is ${{\,\textrm{id}\,}}_A \cdot t^{-1} - \varphi \cdot t^0 :\Phi (A) \rightarrow A$.

Next we explain the key ingredients in the proof of the commutativity of (4.23) and leave it to the reader to figure out the routine to fill in the details based on standard fact about connective *K*-theory such as the Additivity Theorem for Waldhausen categories.

Consider an object $\varphi :\Phi ^k(A) \rightarrow A$ in ${{\,\textrm{Nil}\,}}(\mathcal {A},\Phi ^k)$ given by a nilpotent endomorphism $\varphi :\Phi ^k(A) \rightarrow A$ in $\mathcal {A}$. We have defined its Verschiebung $V_k(\varphi )$ as an object in ${{\,\textrm{Nil}\,}}(A,\Phi )$ given by a specific nilpotent endomorphism

$$\begin{aligned} V_k(\varphi ) :\Phi \bigl (\bigoplus _{j = 0}^{k-1} \Phi ^j(A)\bigr ) =\bigoplus _{i = 1}^k \Phi ^i(A) \rightarrow \bigoplus _{j = 0}^{k-1} \Phi ^j(A) \end{aligned}$$

in $\mathcal {A}$, see (3.2). To it we can assign the morphism

$$\begin{aligned} {{\,\textrm{id}\,}}_{\bigoplus _{j = 0}^{k-1} \Phi ^j(A)} \cdot t^{-1} - V_k(\varphi ) \cdot t^0 :\Phi \bigl (\bigoplus _{j = 0}^{k-1} \Phi ^j(A)\bigr ) =\bigoplus _{i = 1}^k \Phi ^i(A) \rightarrow \bigoplus _{j = 0}^{k-1} \Phi ^j(A) \end{aligned}$$

in $\mathcal {A}_{\Phi }[\mathbb {Z}]$. It is given by the following (*k*, *k*)-matrix

$$\begin{aligned} \begin{pmatrix} {{\,\textrm{id}\,}}_{A} \cdot t^{-1} &  0 &  0 &  \cdots &  0 &  0 &  -\varphi \cdot t^0 \\ -{{\,\textrm{id}\,}}_{\Phi (A)} \cdot t^0 &  {{\,\textrm{id}\,}}_{\Phi (A)} \cdot t^{-1} &  0 &  \cdots &  0 &  0 &  0 \\ 0 &  -{{\,\textrm{id}\,}}_{\Phi ^2(A)} \cdot t^0 &  {{\,\textrm{id}\,}}_{\Phi ^2(A)} \cdot t^{-1} &  \cdots &  0 &  0 &  0 \\ \vdots &  \vdots &  \vdots &  \vdots &  \vdots &  \vdots &  \vdots \\ 0 &  0 &  0 &  \cdots &  -{{\,\textrm{id}\,}}_{\Phi ^{k-3}(A)} \cdot t^{-1} &  0 &  0 \\ 0 &  0 &  0 &  \cdots &  -{{\,\textrm{id}\,}}_{\Phi ^{k-2}(A)} \cdot t^0 &  -{{\,\textrm{id}\,}}_{\Phi ^{k-2}(A)} \cdot t^{-1} &  0 \\ 0 &  0 &  0 &  \cdots &  0 &  -{{\,\textrm{id}\,}}_{\Phi ^{k-1}(A)} \cdot t^0 &  -{{\,\textrm{id}\,}}_{\Phi ^{k-1}(A)} \cdot t^{-1} \end{pmatrix} \end{aligned}$$

of morphisms in $\mathcal {A}_{\Phi }[\mathbb {Z}]$.

On the other hand we can assign to $\varphi :\Phi ^k(A) \rightarrow A$ in $\mathcal {A}$ the morphism in $\mathcal {A}_{\Phi ^k}[\mathbb {Z}]$ given

$$\begin{aligned} {{\,\textrm{id}\,}}_{A} \cdot t^{-1} - \varphi \cdot t^0 :\Phi ^k(A) \rightarrow A. \end{aligned}$$

Its image under the induction functor $(i_k)_*$ is the morphism in $\mathcal {A}_{\Phi }[\mathbb {Z}]$ given by

$$\begin{aligned} {{\,\textrm{id}\,}}_A \cdot t^{-k} - \varphi \cdot t^0 :\Phi ^k(A) \rightarrow A. \end{aligned}$$

Consider the morphism $w :\bigoplus _{i = 1}^k \Phi ^i(A) \xrightarrow {\cong } \bigoplus _{i = 1}^k \Phi ^i(A)$ in $\mathcal {A}_{\Phi }[\mathbb {Z}]$ given by the (*k*, *k*)-matrix

$$\begin{aligned} \begin{pmatrix} {{\,\textrm{id}\,}}_{\Phi (A)} \cdot t^0 &  {{\,\textrm{id}\,}}_{\Phi (A)} \cdot t^{-1} &  {{\,\textrm{id}\,}}_{\Phi (A)} \cdot t^{-2} &  \cdots &  {{\,\textrm{id}\,}}_{\Phi (A)} \cdot t^{-k+3} &  {{\,\textrm{id}\,}}_{\Phi (A)} \cdot t^{-k+2} &  {{\,\textrm{id}\,}}_{\Phi (A)} \cdot t^{-k+1} \\ 0 &  {{\,\textrm{id}\,}}_{\Phi ^2(A)} \cdot t^0 &  {{\,\textrm{id}\,}}_{\Phi ^2(A)} \cdot t^{-1} &  \cdots &  {{\,\textrm{id}\,}}_{\Phi ^2(A)} \cdot t^{-k+4} &  {{\,\textrm{id}\,}}_{\Phi ^2(A)} \cdot t^{-k+3} &  {{\,\textrm{id}\,}}_{\Phi ^2(A)} \cdot t^{-k+2} \\ 0 &  0 &  {{\,\textrm{id}\,}}_{\Phi ^3(A)} \cdot t^0 &  \cdots &  {{\,\textrm{id}\,}}_{\Phi ^3(A)} \cdot t^{-k+5} &  {{\,\textrm{id}\,}}_{\Phi ^3(A)} \cdot t^{-k+4} &  {{\,\textrm{id}\,}}_{\Phi ^3(A)} \cdot t^{-k+3} \\ \vdots &  \vdots &  \vdots &  \vdots &  \vdots &  \vdots &  \vdots \\ 0 &  0 &  0 &  \cdots &  {{\,\textrm{id}\,}}_{\Phi ^{k-2}(A)} \cdot t^0 &  {{\,\textrm{id}\,}}_{\Phi ^{k-2}(A)} \cdot t^{-1} &  {{\,\textrm{id}\,}}_{\Phi ^{k-2}(A)} \cdot t^{-2} \\ 0 &  0 &  0 &  \cdots &  0 &  {{\,\textrm{id}\,}}_{\Phi ^{k-1}(A)} \cdot t^0 &  {{\,\textrm{id}\,}}_{\Phi ^{k-1}(A)} \cdot t^{-1} \\ 0 &  0 &  0 &  \cdots &  0 &  0 &  {{\,\textrm{id}\,}}_{\phi ^{k}(A)} \cdot t^0 \end{pmatrix} \end{aligned}$$

of morphisms in $\mathcal {A}_{\Phi }[\mathbb {Z}]$. Note that *w* is the same as the identity (*k*, *k*)-matrix from the *K*-theoretic point of view by its block structure. Then the composite

$$\begin{aligned} \bigl ({{\,\textrm{id}\,}}_{\bigoplus _{j = 0}^{k-1} \Phi ^j(A)} \cdot t^{-1} - V_k(\varphi ) \cdot t^0\bigr ) \circ w :\Phi \bigl (\bigoplus _{j = 0}^{k-1} \Phi ^j(A)\bigr ) =\bigoplus _{i = 1}^k \Phi ^i(A) \rightarrow \bigoplus _{j = 0}^{k-1} \Phi ^j(A) \end{aligned}$$

is given by the (*k*, *k*)-matrix

$$\begin{aligned} \begin{pmatrix} {{\,\textrm{id}\,}}_A \cdot t^{-1} &  {{\,\textrm{id}\,}}_A \cdot t^{-2} &  {{\,\textrm{id}\,}}_A \cdot t^{-3} &  \cdots &  {{\,\textrm{id}\,}}_A \cdot t^{-k +2} &  {{\,\textrm{id}\,}}_A \cdot t^{-k +1} &  {{\,\textrm{id}\,}}_A \cdot t^{-k} - \varphi \cdot t^0 \\ -{{\,\textrm{id}\,}}_{\Phi (A)} \cdot t^0 &  0 &  0 &  \cdots &  0 &  0 &  0 \\ 0 &  -{{\,\textrm{id}\,}}_{\Phi ^2(A)} \cdot t^0 &  0 &  \cdots &  0 &  0 &  0 \\ \vdots &  \vdots &  \vdots &  \vdots &  \vdots &  \vdots &  \vdots \\ 0 &  0 &  0 &  \cdots &  0 &  0 &  0 \\ 0 &  0 &  0 &  \cdots &  -{{\,\textrm{id}\,}}_{\Phi ^{k-2}(A)} \cdot t^0 &  0 &  0 \\ 0 &  0 &  0 &  \cdots &  0 &  -{{\,\textrm{id}\,}}_{\Phi ^{k-1}(A)} \cdot t^0 &  0 \end{pmatrix} \end{aligned}$$

of morphisms in $\mathcal {A}_{\Phi }[\mathbb {Z}]$. Note that this composite looks like ${{\,\textrm{id}\,}}_A \cdot t^{-k} - \varphi \cdot t^0$ from the *K*-theoretic point of view by its block structure. This finishes the proof of assertion (i).

(ii) The following diagram commutes for $n \in \mathbb {Z}$where the upper horizontal arrow is the isomorphism (4.17) for $\Phi$, the lower horizontal arrow is the isomorphism (4.17) for $\Phi ^k$ and the vertical homomorphism have been defined in (4.7), (4.9), (4.15) and (4.19). The proof of commutativity for the terms $N\!K_n(\mathcal {A}_{\Phi ^k}[t])$ and $N\!K_n(\mathcal {A}_{\Phi ^k}[t^{-1}])$ is obvious, the one for the term $\pi _n(\textbf{T}_{\textbf{K}(\Phi ^{-k})})$ is left to the reader.

Therefore it remains to show that the diagram4.26commutes for $n \in \mathbb {Z}$. By the same argument as it appears in the commutativity of the diagram (4.22), one can show that it suffices to prove the commutativity of (4.26) for $n \ge 1$, or, in other words for connective *K*-theory. By inspecting [29] one sees that this boils down to show that the following diagram4.27commutes for $n \ge 1$.

Next we explain the key ingredients in the proof of the commutativity of (4.27) for connective *K*-theory and leave it to the reader to figure out the routine to fill in the details based on standard fact about *K*-theory such as the Additivity Theorem for Waldhausen categories.

Let $\Phi :\mathcal {A}\xrightarrow {\cong } \mathcal {A}$ be an automorphism of an additive category. Consider a nilpotent endomorphism $\varphi :\Phi (A) \rightarrow A$ representing an element in ${{\,\textrm{Nil}\,}}(\mathcal {A},\Phi )$. Consider the morphism ${{\,\textrm{id}\,}}_A \cdot t^{-1} - \varphi \cdot t^0 :\Phi (A) \rightarrow A$ in $\mathcal {A}_{\Phi }[\mathbb {Z}]$. The functor $i_k^* :\mathcal {A}_{\Phi }[\mathbb {Z}] \rightarrow \mathcal {A}_{\Phi ^k}[\mathbb {Z}]$ sends it to the morphism $\bigoplus _{i = 1}^k \Phi ^i(A) \rightarrow \bigoplus _{j = 0}^{k-1} \Phi ^j(A)$ in $\mathcal {A}_{\Phi ^k}[\mathbb {Z}]$ that is given by the (*k*, *k*)-matrix

$$\begin{aligned} \begin{pmatrix} - \varphi \cdot t^0 &  0 &  0 &  \cdots &  0&  0 &  {{\,\textrm{id}\,}}_{\Phi ^k(A)} \cdot t^{-1} \\ {{\,\textrm{id}\,}}_{\Phi (A)} \cdot t^0 &  - \Phi (\varphi ) \cdot t^0 &  0 &  \cdots &  0 &  0 &  0 \\ 0 &  {{\,\textrm{id}\,}}_{\Phi ^2(A)} \cdot t^0 &  - \Phi ^2(\varphi ) \cdot t^0 &  \cdots &  0 &  0 &  0 \\ \vdots &  \vdots &  \vdots &  \vdots &  \vdots &  \vdots &  \vdots \\ 0 &  0 &  0 &  \cdots &  {{\,\textrm{id}\,}}_{\Phi ^{k-2}(A)} \cdot t^0 &  - \Phi ^{k-2}(\varphi ) \cdot t^0 &  0 \\ 0 &  0 &  0 &  \cdots &  0 &  {{\,\textrm{id}\,}}_{\Phi ^{k-1}(A)} \cdot t^0 &  - \Phi ^{k-1}(\varphi ) \cdot t^0 \end{pmatrix} \end{aligned}$$

of morphisms in $\mathcal {A}_{\Phi }[\mathbb {Z}]$.

If we apply the Frobenius $F_k$ operator to the object $\varphi :\Phi (A) \rightarrow A$ of ${{\,\textrm{Nil}\,}}(\mathcal {A},\Phi )$, we obtain the object $\varphi ^{(k)} :\Phi ^k(A) \xrightarrow {\Phi ^{k-1}(\varphi )} \Phi ^{k-1}(A) \xrightarrow {\Phi ^{k-2}(\varphi )} \cdots \xrightarrow {\varphi } A$ of ${{\,\textrm{Nil}\,}}(\mathcal {A},\Phi ^k)$. To it we can assign the morphism ${{\,\textrm{id}\,}}_A \cdot t^{-1} - \varphi ^{(k)} \cdot t^0 :\Phi ^k(A) \rightarrow A$ in $\mathcal {A}_{\Phi ^k}[\mathbb {Z}]$.

Consider the morphism

$$\begin{aligned} u :\bigoplus _{i = 1}^k \Phi ^i(A) \rightarrow \bigoplus _{j= 0}^{k-1} \Phi ^j(A) \end{aligned}$$

in $\mathcal {A}_{\Phi ^k}[\mathbb {Z}]$ that is given by the (*k*, *k*)-matrix

$$\begin{aligned} \begin{pmatrix} - \varphi \cdot t^0 &  - \varphi ^{(2)} \cdot t^0 &  - \varphi ^{(3)} \cdot t^0 &  \cdots &  - \varphi ^{(k-2)} \cdot t^0 &  - \varphi ^{(k-1)} \cdot t^0 &  {{\,\textrm{id}\,}}_A \cdot t^{-1} - \varphi ^{(k)} \cdot t^0 \\ {{\,\textrm{id}\,}}_{\Phi (A)} \cdot t^0 &  0 &  0 &  \cdots &  0 &  0 &  0 \\ 0 &  {{\,\textrm{id}\,}}_{\Phi ^2(A)} \cdot t^0 &  0 &  \cdots &  0 &  0 &  0 \\ 0 &  0 &  {{\,\textrm{id}\,}}_{\Phi ^3(A)} \cdot t^0 &  \cdots &  0 &  0 &  0 \\ \vdots &  \vdots &  \vdots &  \vdots &  \vdots &  \vdots &  \vdots \\ 0 &  0 &  0 &  \cdots &  {{\,\textrm{id}\,}}_{\Phi ^{k-2}(A)} \cdot t^0 &  0 &  0 \\ 0 &  0 &  0 &  \cdots &  0 &  {{\,\textrm{id}\,}}_{\Phi ^{k-1}(A)} \cdot t^0 &  0 \end{pmatrix} \end{aligned}$$

of morphisms in $\mathcal {A}_{\Phi ^k}[\mathbb {Z}]$. Note that from a *K*-theoretic point of view this morphism should give the same element in *K*-theory as the morphisms ${{\,\textrm{id}\,}}_A \cdot t^{-1} - \varphi ^{(k)} \cdot t^0 :\Phi ^k(A) \rightarrow A$ in $\mathcal {A}_{\Phi ^k}[\mathbb {Z}]$, just view its special block structure.

We also have the automorphism

$$\begin{aligned} v :\bigoplus _{i = 1}^{k} \Phi ^j(A) \xrightarrow {\cong } \bigoplus _{j = 1}^{k} \Phi ^j(A) \end{aligned}$$

in $\mathcal {A}_{\Phi ^k}[\mathbb {Z}]$ given by the (*k*, *k*)-matrix

$$\begin{aligned} \begin{pmatrix} {{\,\textrm{id}\,}}_{\Phi (A)} \cdot t^0 &  - \Phi (\varphi ) \cdot t^0 &  0 &  \cdots &  0 &  0 &  0 \\ 0 &  {{\,\textrm{id}\,}}_{\Phi ^2(A)} \cdot t^0 &  - \Phi ^2(\varphi ) \cdot t^0 &  \cdots &  0 &  0 &  0 \\ 0 &  0 &  - {{\,\textrm{id}\,}}_{\Phi ^3(A)} \cdot t^0 &  \cdots &  0 &  0 &  0 \\ \vdots &  \vdots &  \vdots &  \vdots &  \vdots &  \vdots &  \vdots \\ 0 &  0 &  0 &  \cdots &  {{\,\textrm{id}\,}}_{\Phi ^{k-2}(A)} \cdot t^0 &  - \Phi ^{k-2}(\varphi ) \cdot t^0 &  0 \\ 0 &  0 &  0 &  \cdots &  0 &  {{\,\textrm{id}\,}}_{\Phi ^{k-1}(A)} \cdot t^0 &  \Phi ^{k-1}(\varphi ) \cdot t^0 \\ 0 &  0 &  0 &  \cdots &  0 &  0 &  {{\,\textrm{id}\,}}_{\Phi ^{k}(A)} \cdot t^0 \end{pmatrix} \end{aligned}$$

of morphisms in $\mathcal {A}_{\Phi ^k}[\mathbb {Z}]$. Note that *v* is the same as the identity (*k*, *k*)-matrix from the *K*-theoretic point of view by its block structure.

Now one easily check the equality of morphisms $\bigoplus _{i = 1}^k \Phi ^i(A) \rightarrow \bigoplus _{i = 0}^{k} \Phi ^j(A)$ in $\mathcal {A}_{\Phi ^k}[\mathbb {Z}]$

$$\begin{aligned} u \circ v = i_k^*\bigl ({{\,\textrm{id}\,}}_A \cdot t^{-1} - \varphi \cdot t^0\bigr ). \end{aligned}$$

Now Theorem 4.16 follows. $\square$

Let

4.28 $$\begin{aligned} \overline{s}_n :\overline{K}_n({{\,\textrm{Nil}\,}}(\mathcal {A},\Phi ))\rightarrow &   K_{n-1}(\mathcal {A}_{\Phi }[\mathbb {Z}]) \end{aligned}$$

be the homomorphism coming from the Bass-Heller-Swan homomorphism, see (4.5), and the inclusion of the first Nil-term. Let

4.29 $$\begin{aligned} \overline{r}_n :K_{n-1}(\mathcal {A}_{\Phi }[\mathbb {Z}])\rightarrow &   \overline{K}_n({{\,\textrm{Nil}\,}}(\mathcal {A},\Phi )) \end{aligned}$$

be the homomorphisms coming from the Bass–Heller Swan homomorphism, see (4.5), and the projection onto the first Nil-term. Obviously we have $\overline{r}_n \circ \overline{s}_n = {{\,\textrm{id}\,}}$.

Theorem 4.16 implies

### Corollary 4.30

(i) We have for every $n \in \mathbb {Z}$ the commutative diagram  where the vertical arrows have been defined n (4.28) and the horizontal arrows have been defined in (4.12) and (4.14);
(ii) We have for every $n \in \mathbb {Z}$ the commutative diagram  where the vertical arrows have been defined in (4.29) and the horizontal arrows have been defined in (4.13) and (4.15).

Corollary 4.30 has already been proved for commutative rings as coefficients in the untwisted case by Stienstra [35, Theorem 4.7].

For a natural number *D*, let ${{\,\textrm{Nil}\,}}(\mathcal {A},\Phi )_D$ be the full subcategory of ${{\,\textrm{Nil}\,}}(\mathcal {A},\Phi )$ consisting of objects $(P,\varphi )$ satisfying $\varphi ^{(D)} = 0$. Obviously ${{\,\textrm{Nil}\,}}(\mathcal {A},\Phi ) = \bigcup _{d \ge 0 } {{\,\textrm{Nil}\,}}(\mathcal {A},\Phi )_d$. Hence the canonical maps4.314.32are bijective. Given an element $z \in \overline{K}_n({{\,\textrm{Nil}\,}}(\mathcal {A},\Phi ))$ and a natural number *D*, we say that *z* is of *nilpotence degree* $\le D$ if *z* lies in the image of $\overline{K_n}({{\,\textrm{Nil}\,}}(\mathcal {A},\Phi )_D)\rightarrow \overline{K}_n({{\,\textrm{Nil}\,}}(\mathcal {A},\Phi ))$.

The next result is known for rings as coefficient in the untwisted case, see Farrell [19, Lemma 3] for $n = 1$, Grunewald [20, Prop. 4.6], Stienstra [35, p. 90], and Weibel [41, p. 479].

### Lemma 4.33

Fix an integer $n \in \mathbb {Z}$ Consider an element $z \in \overline{K}_n({{\,\textrm{Nil}\,}}(\mathcal {A},\Phi ))$ of nilpotence degree $\le D$.

Then the composite

$$\begin{aligned} \overline{K}_{n-1}({{\,\textrm{Nil}\,}}(\mathcal {A},\Phi )) \xrightarrow {\overline{s}_n} K_n(\mathcal {A}_{\Phi }[\mathbb {Z}]) \xrightarrow {K_n(i_k^*)} K_n(\mathcal {A}_{\Phi ^k}[\mathbb {Z}]) \end{aligned}$$

sends *z* for every $k \ge D$ to zero, where $\overline{s}_n$ and $K_n(i_k^*)$ have been defined in (4.28) and (4.15).

### Proof

Since the composite ${{\,\textrm{Nil}\,}}(\mathcal {A},\Phi )_D\rightarrow {{\,\textrm{Nil}\,}}(\mathcal {A},\Phi ) \xrightarrow {F_k} {{\,\textrm{Nil}\,}}(\mathcal {A},\Phi ^k)$ sends an object $(P,\phi )$ to (*P*, 0) for $k \ge D$, the composite $\overline{K}_n({{\,\textrm{Nil}\,}}(\mathcal {A},\Phi )) \xrightarrow {\overline{K}_n(F_k)} \overline{K}_n({{\,\textrm{Nil}\,}}(\mathcal {A},\Phi ^k))$ sends *z* to zero for $k \ge D$. Now Lemma 4.33 follows from Corollary 4.30 (i). $\square$

## Mackey and Green functors for finite groups

For the reader’s convenience we recall some basics about Mackey and Green functors and Dress induction following [3, Section 2 and 3]. Throughout this section *F* is a finite group.

### Mackey functors

Let  be the category, whose objects are finite *F*-sets and whose morphisms are *F*-maps.

Let $\Lambda$ be an associative commutative ring with unit. Denote by  the abelian category of $\Lambda$-modules. A *bi-functor* $M = (M_*,M^*)$ from  to  consists of a covariant functorand a contravariant functorthat agree on objects. We often write $M(X) = M_*(X) = M^*(X)$ for an object *X* in  and $f_* = M_*(f)$ and $f^* = M^*(f)$ for a morphism $f :X \rightarrow Y$ in 

#### Definition 5.1

(*Mackey functor*) A *Mackey functor M* *for* *F* *with values in* $\Lambda$*-modules* is a bifunctor from  to  such that

- Double Coset formula
  For any cartesian square in  the following diagram of functors of abelian groups commutes
- Additivity
  Consider two objects *X* and *Y* in . Let $i :X \rightarrow X \amalg Y$ and $j:Y \rightarrow X \amalg Y$ be the inclusions. Then the map
  $$\begin{aligned} i^* \times j^* :M(X \amalg Y) \rightarrow M(X) \times M(Y) \end{aligned}$$
  is bijective;

#### Remark 5.2

One easily checks that the condition Additivity implies $M(\emptyset ) = 0$ and is equivalent to the requirement that $M_*(i) \oplus M_*(j) :M(X) \oplus M(Y) \rightarrow M(X \amalg Y)$ is bijective, since the double coset formula implies that ${{\,\textrm{id}\,}}\times {{\,\textrm{id}\,}}:M(\emptyset ) \rightarrow M(\emptyset ) \times M(\emptyset )$ is an isomorphism and that $(M^*(i) \times M^*(j )) \circ (M_*(i) \oplus M_*(j))$ is the identity.

Let *M*, *N* and *L* be bi-functors from  values in $\Lambda$-modules. A pairing

5.3 $$\begin{aligned} M \times N \rightarrow L \end{aligned}$$

is a family of $\Lambda$-bilinear maps

$$\begin{aligned} \mu (X) :M(X) \times N(X) \rightarrow L(X), \quad (m,n) \mapsto \mu (m,n) = m \cdot n \end{aligned}$$

indexed by the objects *X* of  such that for every morphism $f :X \rightarrow Y$ in  we have

5.4 $$\begin{aligned}&\begin{array}{lllll} L^*(f)(x \cdot y) &  = &  M^*(f)(x) \cdot N^*(f)(y), &  &  x \in M(Y), y \in N(Y); \\ x \cdot N_*(f)(y) &  = &  L_*(f)(M^*(f)(x) \cdot y), &  &  x \in M(Y), y \in N(X); \\ M_*(f)(x) \cdot y &  = &  L_*(f)(x \cdot N^*(f)(y)), &  &  x \in M(X), y \in N(Y). \end{array}&\end{aligned}$$

### Green functors

#### Definition 5.5

(*Green functor*) A *Green functor* for the finite group *F* with values in $\Lambda$-modules is a Mackey functor *U* together with a pairing

$$\begin{aligned} \mu :U \times U \rightarrow U \end{aligned}$$

and a choice of elements $1_X \in U(X)$ for each object *X* in  such that for every object *X* in  the pairing $\mu (X) :U(X) \times U(X) \rightarrow U(X)$ and the element $1_X$ determine the structure of an associative $\Lambda$-algebra with unit on *U*(*X*). Moreover, it is required that $U^*(f)(1_Y) = 1_X$ for every morphism $f :X \rightarrow Y$ in .

A (left) *U*-module *M* is a Mackey functor for the group *F* with values in $\Lambda$-modules together with a pairing

$$\begin{aligned} \nu :U \times M \rightarrow M \end{aligned}$$

such that for every object *X* in  the pairing $\nu (X) :U(X) \times M(X) \rightarrow M(X)$ defines the structure of a *U*(*X*)-module on *M*(*X*), where $1_X$ acts as ${{\,\textrm{id}\,}}_{M(X)}$.

### Dress induction

Let *F* be a finite group. Let  be a family of subgroups of *F* that is closed under taking subgroups and conjugation. An example for  is the family $\mathcal {H}_p$ of *p*-hyperelementary subgroups of *F* for any prime *p*. A Green functor $\mathcal {U}$ over *F* is called -*computable* if the canonical $\Lambda$-mapis surjective, where ${{\,\textrm{pr}\,}}_H :F/H \rightarrow F/F$ is the projection. The next result is a mild generalization of the fundamental work of Dress on induction, see [17, 18].

#### Lemma 5.6

Let $\mathcal {U}$ be a Green functor over *F* that is -computable. Consider any $\mathcal {U}$-module $\mathcal {M}$. Then for every element $z \in \mathcal {M}(F/F)$ we can find elements $u_H \in \mathcal {U}(F/H)$ for  satisfyingwhere ${{\,\textrm{pr}\,}}_H :F/H \rightarrow F/F$ is the projection.

#### Proof

By assumption we can write the unit $1_{F/F} \in \mathcal {U}(F/F)$ as the sumfor appropriate elements $u_H \in M(F/H)$. Now we compute using the various axioms for the structure of a Green functor and a module over a Green functor$\square$

### The Green functor ${{\,\textrm{Sw}\,}}_F$

Let *G* be a group. Next we define the *Swan ring* ${{\,\textrm{Sw}\,}}(G)$ associated to *G*. As an abelian group ${{\,\textrm{Sw}\,}}(G)$ is defined in terms of generators and relations as follows. Generators [*M*] are given by $\mathbb {Z}G$-isomorphism classes of $\mathbb {Z}G$-modules *M*, which are finitely generated free as $\mathbb {Z}$-modules. If we have an exact sequence $0 \rightarrow M_0 \rightarrow M_1 \rightarrow M_2 \rightarrow 0$ of such modules, then we require the relation $[M_1] = [M_0] + [M_2]$. The multiplication in the Swan ring is given by the tensor product over $\mathbb {Z}$ with the diagonal *G*-action. Note that ${{\,\textrm{Sw}\,}}(G)$ is a commutative ring, whose unit is given by $\mathbb {Z}$ equipped with the trivial *G*-action.

In [3, Section 3] the definition of ${{\,\textrm{Sw}\,}}(G)$ is extended from groups to small groupoids. For a small groupoid $\mathcal {G}$ an element in ${{\,\textrm{Sw}\,}}(\mathcal {G})$ is given by a covariant functor from $\mathcal {G}$ to the category of finitely generated free abelian groups. Actually, ${{\,\textrm{Sw}\,}}(\mathcal {G})$ is the Grothendieck group of such functors under the obvious objectwise notion of a short exact sequence of such functors. The structure of a commutative ring comes from the tensor product of abelian groups applied objectwise. The unit is represented by the constant functor, which sends every object in $\mathcal {G}$ to $\mathbb {Z}$ and every morphism in $\mathcal {G}$ to ${{\,\textrm{id}\,}}_{\mathbb {Z}}$.

Given a *G*-set *X*, denote by $\mathcal {T}^G(X)$ its *transport groupoid*. Its set of objects is *X*, its set of morphism from $x_0 \rightarrow x_1$ is $G_{x_0,x_1} = \{g \in G \mid x_1 = gx_0\}$, and composition comes from the multiplication in *G*. Now we can consider ${{\,\textrm{Sw}\,}}(\mathcal {T}^G(X))$ for a *G*-set *X*. For every subgroup *H* of *G* there is an obvious isomorphism

5.7 $$\begin{aligned} {{\,\textrm{Sw}\,}}(H) \xrightarrow {\cong } {{\,\textrm{Sw}\,}}(\mathcal {T}^G(G/H)) \end{aligned}$$

coming from the facts that $\mathcal {T}^G(G/H)$ is a connected groupoid and the automorphism group of *eH* is *H*.

The elementary proof that we get for a finite group *F* a Green functor ${{\,\textrm{Sw}\,}}^F$, which is given for a finite *F*-set *X* by ${{\,\textrm{Sw}\,}}^F(X) = {{\,\textrm{Sw}\,}}(\mathcal {T}^F(X))$, can be found in [3, Section 3].

#### Lemma 5.8

Let *F* be a finite group. Then the Green functor with values in $\mathbb {Z}_{(p)}$-modules $\mathbb {Z}_{(p)} \otimes _{\mathbb {Z}} {{\,\textrm{Sw}\,}}_F$ is computable for $\mathcal {H}_p$.

#### Proof

See for instance [3, Proof of Lemma 4.1 (c)] which is based on [38, 6.3.3], [36, Lemma 4.1] and [37, Section 12]. $\square$

Let *p* be a prime and denote by $\mathcal {H}_p$ be the family of *p*-hyperelementary subgroups of the finite group *F*. We get from Lemmas 5.6 and 5.8

#### Lemma 5.9

Let *F* be a finite group. Consider any ${{\,\textrm{Sw}\,}}_F$-module $\mathcal {M}$. Then for every element $z \in \mathcal {M}(F/F)$ we can find elements $a_H \in \mathbb {Z}_{(p)}$ and $u_H \in {{\,\textrm{Sw}\,}}(H)$ for every  such thatholds in $\mathcal {M}(F/F)_{(p)} = \mathbb {Z}_{(p)} \otimes _{\mathbb {Z}} \mathcal {M}(F/F)$, where ${{\,\textrm{pr}\,}}_H :F/H \rightarrow F/F$ is the projection.

#### Remark 5.10

There is also a version of ${{\,\textrm{Sw}\,}}(H)$ for groups *H* and ${{\,\textrm{Sw}\,}}(\mathcal {G})$ for groupoids $\mathcal {G}$, where one replaces finitely generated free abelian group by finitely generated abelian group. These two versions are isomorphic, see [32, page 890] and [3, Section 3] for groupoids.

It is more convenient to work with the version for finitely generated free abelian groups, since for finitely generated free abelian groups *M* the functor $M \otimes _{\mathbb {Z}} -$ is exact.

## *K*-theoretic functors associated to *G*-$\mathbb {Z}$-categories

Let *G* be a group and let $\Lambda$ be a commutative ring.

### The *K*-theoretic covariant -spectrum associated to a *G*-$\mathbb {Z}$-category

Let $\mathcal {A}$ be a *G*-$\Lambda$-category. For a *G*-set *X* we define a $\Lambda$-category $\mathcal {A}(X)$ as follows. Objects are pairs (*A*, *x*) with $A \in {{\,\textrm{ob}\,}}(\mathcal {A})$ and $x \in X$. A morphism $\varphi :(A,x) \rightarrow (A',x')$ is a formal finite sum $\varphi = \sum _{g \in G_{x,x'}} \varphi _g \cdot g$, where $G_{x,x'} = \{g \in G \mid x' = gx\}$ and $\varphi _g$ is a morphism in $\mathcal {A}$ from *gA* to $A'$. For a morphism $\varphi ' :(A',x) \rightarrow (A'',x'')$ given by the formal finite sum $\varphi ' = \sum _{g' \in G_{x',x''}} \varphi '_g \cdot g'$, we define the composite $\varphi ' \circ \varphi :(A,x) \rightarrow (A'',x'')$ by the finite formal sum $\varphi ' \circ \varphi = \sum _{g'' \in G_{x,x''}} (\varphi ' \circ \varphi )_{g''} \cdot g''$, where for $g'' \in G_{x,x''}$ we put

$$\begin{aligned} (\varphi ' \circ \varphi )_{g''} = \sum _{\begin{array}{c} g \in G_{x,x'}, g' \in G_{x',x''} \\ g'' = g'g \end{array}} \varphi '_{g'} \circ g'\varphi _g. \end{aligned}$$

Given a *G*-map $f :X \rightarrow Y$, define a functor of $\Lambda$-categories $\mathcal {A}(f) :\mathcal {A}(X) \rightarrow \mathcal {A}(Y)$ by sending an object (*A*, *x*) to the object (*A*, *f*(*x*)) and a morphism $\varphi :(A,x) \rightarrow (A',x')$ given by the formal sum $\varphi = \sum _{g \in G_{x,x'}} \varphi _g \cdot g$ to the morphism given the same finite formal sum. This makes sense because of $G_{x,x'} \subseteq G_{f(x),f(x')}$. Hence we get a covariant functor6.1It induces the covariant functor6.2In particular we get a covariant functor called *the* *K*-*theoretic covariant *-*spectrum associated to* $\mathcal {A}$6.3

#### Remark 6.4

Let $H_*^G(-;\textbf{K}_{\mathcal {A}})$ be the *G*-homology theory associated to $\textbf{K}_{\mathcal {A}}$. It has the property that $H_n^G(G/H;\textbf{K}_{\mathcal {A}}) = \pi _n(\textbf{K}_{\mathcal {A}}(G/H))$ holds for any subgroup *H* of *G*. Denote by $E_{{{{\mathcal {F}}}\textrm{in}}}(G)$ and $E_{{{{\mathcal {V}}}\textrm{cyc}}}(G)$ respectively the classifying space for the family ${{{\mathcal {F}}}\textrm{in}}$ of finite subgroups and of the family ${{{\mathcal {V}}}\textrm{cyc}}$ of virtually cyclic subgroups of *G*, see for instance [23]. The projection $E_{{{{\mathcal {V}}}\textrm{cyc}}}(G) \rightarrow G/G$ induces the assembly map for $n \in \mathbb {Z}$

$$\begin{aligned} H_n^G(E_{{{{\mathcal {V}}}\textrm{cyc}}}(G);\textbf{K}_{\mathcal {A}}) \rightarrow H_n^G(G/G;\textbf{K}_{\mathcal {A}}) = \pi _n(\textbf{K}_{\mathcal {A}}(G/G)). \end{aligned}$$

The Farrell–Jones Conjecture predicts that it is bijective for all $n \in \mathbb {Z}$. If *R* is a ring and we take for $\mathcal {A}$ the additive category $\underline{R}_{\oplus }$, then this assembly map can be identified with the assembly map 1.4.

The canoncial map $E_{{{{\mathcal {F}}}\textrm{in}}}(G) \rightarrow E_{{{{\mathcal {V}}}\textrm{cyc}}}(G)$ induces the relative assembly map

$$\begin{aligned} H_n^G(E_{{{{\mathcal {F}}}\textrm{in}}}(G);\textbf{K}_{\mathcal {A}}) \rightarrow H_n^G(E_{{{{\mathcal {V}}}\textrm{cyc}}}(G);\textbf{K}_{\mathcal {A}}). \end{aligned}$$

If *R* is a ring and we take for $\mathcal {A}$ the additive category $\underline{R}_{\oplus }$, then this assembly map can be identified with the relative assembly map appearing in Theorem 1.2.

For more information about assembly maps and the Farrell–Jones Conjecture we refer for instance to [24–26].

### Restriction

For a *G*-map $f :X \rightarrow Y$, we think of $K_n(\mathcal {A}(f)_{\oplus }) :K_n(\mathcal {A}(X)_{\oplus }) \rightarrow K_n(\mathcal {A}(Y)_{\oplus })$ as induction. We can define a kind of restriction, if we assume that $f^{-1}(y)$ is finite for every $y\in Y$, as follows. Next we define a functor of $\Lambda$-categories

$$\begin{aligned} {{\,\textrm{res}\,}}(f) :\mathcal {A}(Y) \rightarrow \mathcal {A}(X)_{\oplus }. \end{aligned}$$

It sends an object (*A*, *y*) to $\bigoplus _{x \in f^{-1}(y)} (A,x)$. Consider a morphism $\varphi :(A,y) \rightarrow (A',y')$ given by a formal sum $\sum _{g \in G_{y,y'}} \varphi _g \cdot g$. Then ${{\,\textrm{res}\,}}(f)(\varphi ) :\bigoplus _{x \in f^{-1}(y)} (A,x) \rightarrow \bigoplus _{x' \in f^{-1}(y')} (A',x')$ is given by the collection of morphisms ${{\,\textrm{res}\,}}(f)(\varphi )_{x,x'} :(A,x) \rightarrow (A',x')$ for $x \in f^{-1}(y)$ and $x' \in f^{-1}(y')$ if we put ${{\,\textrm{res}\,}}(f)(\varphi )_{x,x'} = \sum _{g \in G_{x,x'}} \varphi _g \cdot g$. This makes sense because of $G_{x,x'} \subseteq G_{y,y'}$. The functor ${{\,\textrm{res}\,}}(f)$ induces a functor of additive $\Lambda$-categories ${{\,\textrm{res}\,}}(f)_{\oplus } :\mathcal {A}(Y)_{\oplus } \rightarrow (\mathcal {A}(X)_{\oplus })_{\oplus }$. Composing it with the obvious equivalence of additive $\Lambda$-categories $(\mathcal {A}(X)_{\oplus })_{\oplus } \xrightarrow {\simeq } \mathcal {A}(X)_{\oplus }$ yields a functor of additive $\Lambda$-categories, denoted in the same way

6.5 $$\begin{aligned} {{\,\textrm{res}\,}}(f)_{\oplus } :\mathcal {A}(Y)_{\oplus } \rightarrow \mathcal {A}(X)_{\oplus }. \end{aligned}$$

### The pairing with the Swan group

In this section we construct a bilinear pairing for a *G*-set *X* and $n \in \mathbb {Z}$

6.6 $$\begin{aligned} P^G_X :{{\,\textrm{Sw}\,}}(\mathcal {T}^G(X)) \times K_n(\mathcal {A}(X)_{\oplus }) \rightarrow K_n(\mathcal {A}(X)_{\oplus }). \end{aligned}$$

We have to construct for any covariant functor *M* from $\mathcal {T}^G(X)$ to the category of finitely generated free abelian groups a functor of $\mathbb {Z}$-categories $F(M) :\mathcal {A}(X) \rightarrow \mathcal {A}(X)_{\oplus }$, since then we can define for the element $[M] \in {{\,\textrm{Sw}\,}}(\mathcal {T}^G(X))$ represented by *M* the homomorphism $P([M],-) :K_n(\mathcal {A}(X)_{\oplus }) \rightarrow K_n(\mathcal {A}(X)_{\oplus })$ to be $K_n(F(M)_{\oplus })$. For $x \in X$ let *r*(*x*) be the rank of the finitely generated free abelian group *M*(*x*) and choose an ordered basis $\{b_1, \ldots , b_{r(x)}\}$ of *M*(*x*). Consider $x,x' \in X$ with $G_{x,x'} \not = 0$. Then $r(x) = r(x')$ and for $g \in G_{x,x'}$ we get an invertible (*r*(*x*), *r*(*x*))-matrix with entry $\rho (g)_{i,i'} \in \mathbb {Z}$ for $i \in \{1, \ldots , r(x)\}$ and $i' \in \{1, \ldots , r(x')\}$, which describes the homomorphism of finitely generated free abelian groups $M(g) :M(x) \rightarrow M(x')$ for the morphism $g :x \rightarrow x'$ in $\tau ^G(X)$ with respect to the chosen ordered bases. Now *F*(*M*) sends an object (*A*, *x*) to $\bigoplus _{i =1 }^{r(x)} A(x)$. A morphism $\sum _{g \in G} f_g \cdot g :(A,x) \rightarrow (A',x')$ is sent to the morphism $\bigoplus _{i =1 }^{r(x)} A(x) \rightarrow \bigoplus _{i' =1 }^{r(x')} A'(x')$, whose component for $i \in \{1, \ldots , r(x)\}$ and $i' \in \{1, \ldots , r(x')\}$ is given by $\sum _{g \in G} \rho (g)_{i,i'} \cdot f_g \cdot g :(A,x) \rightarrow (A',x')$. Note that the choice of basis does not matter, since it does not change the equivalence class of *F*(*M*). We leave it to the reader to check that we get a well-defined bilinear pairing (6.6).

### The special case of a ring as coefficients

The following example is illuminating and the most important one.

Let $\Lambda$ be a commutative ring and let *R* be a $\Lambda$-algebra, where we tacitly always require that $\lambda r = r \lambda$ holds for every $\lambda \in \Lambda$ and $r \in R$. Consider a group homomorphism $\rho :G \rightarrow {{\,\textrm{aut}\,}}_{\Lambda }(R)$ to the group of automorphisms of the $\Lambda$-algebra *R*. The *twisted group ring with* *R-coefficients* $R_{\rho }[G]$ is defined as follows. Elements are finite formal sums $\sum _{g \in G} r_g \cdot g$ for $r_g \in R$. The multiplication in $R_{\rho }[G]$ is given by

$$\begin{aligned} \left( \sum _{g \in G} r_g \cdot g\right) \cdot \left( \sum _{g' \in G} r'_{g'} \cdot g'\right) = \sum _{g'' \in G} \left( \sum _{\begin{array}{c} g,g' \in G\\ g'' = g'g \end{array}} r_g \cdot \rho (g)(r'_{g'})\right) \cdot g''. \end{aligned}$$

Note that $R_{\rho }[G]$ inherits the structure of $\Lambda$-algebra from *R*.

Define $\underline{R}$ to be the $\Lambda$-category, which has precisely one object $*_R$ and whose $\Lambda$-module of endomorphisms is *R*. Composition is given by the multiplication in *R* and the $\Lambda$-structure on the endomorphisms comes from the structure of a $\Lambda$-algebra. From $\rho$ we obtain the structure of *G*-$\Lambda$-category on $\underline{R}$ and we can consider the $\Lambda$-category $\underline{R}(X)$ for any *G*-set *X*.

Consider a subgroup $H \subseteq G$. There is an equivalence of $\Lambda$-categories

6.7 $$\begin{aligned} T(H) :\underline{R_{\rho |_H}[H]} \xrightarrow {\simeq } \underline{R}(G/H), \end{aligned}$$

that sends the object $*_{R_{\rho |_H}[H]}$ to the object $(*_R, eH)$ and a morphism in $\underline{R_{\rho |_H}[H]}$, which is given by the element $\sum _{h \in H} r_h \cdot h$ in $R_{\rho |_H}[H]$, to the morphism $(*_R, eH) \rightarrow (*_R, eH)$ in $\underline{R}(G/H)$ given by $\sum _{h \in H} r_h \cdot h$ again. Obviously *T*(*H*) is fully faithful. Every object in $\underline{R}(G/H)$ is isomorphic to an object in the image of *T*(*H*), namely, for any object $(*_R, gH)$ we get an isomorphism $(*_R, eH) \xrightarrow {\cong } (*_R, gH)$ by $1_R \cdot g$. Hence $T_H$ is an equivalence of $\Lambda$-categories. From (6.7) we obtain an equivalence of additive $\Lambda$-categories

6.8 $$\begin{aligned} T(H)_{\oplus } :\underline{R_{\rho |_H}[H]}_{\oplus } \xrightarrow {\simeq } \underline{R}(G/H)_{\oplus }. \end{aligned}$$

and hence for every $n \in \mathbb {Z}$ an isomorphism

6.9 $$\begin{aligned} K_n(T(H)_{\oplus }) :K_n(R_{\rho |_H}[H]) \xrightarrow {\cong } K_n(\underline{R}(G/H)_{\oplus }). \end{aligned}$$

Consider subgroups $H \subseteq K \subseteq G$ with $[K:H] < \infty$. Let ${{\,\textrm{pr}\,}}:G/H \rightarrow G/K$ be the projection and $i :H \rightarrow K$ be the inclusion. Then $K_n(\underline{R}({{\,\textrm{pr}\,}})) :K_n(\underline{R}(G/H)) \rightarrow K_n(\underline{R}(G/K))$ corresponds under the isomorphism (6.9) to the induction homomorphism $i_* :K_n(R_{\rho |_H}[H]) \rightarrow K_n(R_{\rho |_K}[K])$, whereas $K_n({{\,\textrm{res}\,}}({{\,\textrm{pr}\,}})_{\oplus }) :K_n(\underline{R}(G/K)_{\oplus }) \rightarrow K_n(\underline{R}(G/H)_{\oplus })$ corresponds under the isomorphism (6.9) to the restriction homomorphism $i^* :K_n(R_{\rho |_K}[K]) \rightarrow K_n(R_{\rho |_H}[H])$. The pairing *P* of (6.6) corresponds under the isomorphisms (5.7) and (6.9) to the pairing ${{\,\textrm{Sw}\,}}(H) \times K_n(R_{\rho |_H}[H]) \rightarrow K_n(R_{\rho |_H}[H])$ coming from the fact that, for a $\mathbb {Z}H$-module *M*, whose underlying abelian group is finitely generated free, and a finitely generated projective $R_{\rho |_H}[H]$-module *P*, we can equip $M \otimes _{\mathbb {Z}} P$ with the $R_{\rho |_H}[H]$-module structure given by

$$\begin{aligned} \left( \sum _{g \in G} r_h \cdot h\right) \cdot (m \otimes p) = \sum _{h \in H} \bigl (hm \otimes (r_h \cdot h \cdot p)\bigr ) \end{aligned}$$

for $\sum _{h \in H} r_h \cdot h \in R_{\rho |_H}[H]$, $m \in M$, and $p \in P$ and thus obtain a finitely generated projective $R_{\rho |_H}[H]$-module.

### The Green and Mackey structure of a finite quotient group

Fix a (not necessarily finite) group *G* and a surjective group homomorphism $\nu :G \rightarrow F$ onto a finite group *F*. Let $\mathcal {A}$ be a *G*-$\mathbb {Z}$-category. Fix $n \in \mathbb {Z}$. Then we can define a module over the Green functor ${{\,\textrm{Sw}\,}}_F$ for *F* as follows. The underlying Mackey functor $M(Z,n) = (M(Z,n)_*,M(Z,n)^*)$ is on an object *X* which is a finite *F*-set *X*, given by

$$\begin{aligned} M(Z,n)_*(X) = M(Z,n)^*(X) = K_n(\mathcal {A}(\nu ^*X)_{\oplus }), \end{aligned}$$

where $\nu ^*X$ is the *G*-set obtained from the *F*-set *X* by restriction with $\nu$. The covariant functor $M(Z,n)_*$ sends an *F*-map $f :X \rightarrow Y$ to the homomorphism $K_n(\mathcal {A}(\nu ^*f)_{\oplus }) :K_n(\mathcal {A}(\nu ^*X)_{\oplus }) \rightarrow K_n(\mathcal {A}[\nu ^*Y]_{\oplus })$ defined in (6.2). The contravariant functor $M(Z,n)_*$ sends an *F*-map $f :X \rightarrow Y$ to $K_n({{\,\textrm{res}\,}}(\nu ^*f)_{\oplus }) :K_n(\mathcal {A}[\nu ^*Y]_{\oplus }) \rightarrow K_n(\mathcal {A}(\nu ^*X)_{\oplus })$ for the functor of additive categories ${{\,\textrm{res}\,}}(\nu ^*f)_{\oplus }$ defined in (6.5). For a finite *F*-set *X* we get a map of abelian groups

6.10 $$\begin{aligned} \nu ^* :{{\,\textrm{Sw}\,}}_F(X) \rightarrow {{\,\textrm{Sw}\,}}_G(\nu ^*X) \end{aligned}$$

by precomposing a covariant functor *M*(*Z*, *n*) from $\tau ^F(X)$ to the category of finitely generated free abelian groups with the obvious functor $\mathcal {T}^G(\nu ^*X) \rightarrow \mathcal {T}^F(X)$ induced by $\nu$. Now we obtain from (6.6) and (6.10) for all finite *F*-sets *X* a pairing

$$\begin{aligned} {{\,\textrm{Sw}\,}}_F(X) \otimes K_n(\mathcal {A}(\nu ^*X)_{\oplus }) \rightarrow K_n(\mathcal {A}(\nu ^*X)_{\oplus }). \end{aligned}$$

We leave the lengthy but straightforward proof to the reader that these data define a Mackey functor $\mathcal {M}$ for *F* and the structure of a Green-module over ${{\,\textrm{Sw}\,}}_F$ on it. This fact is not surprising in view of Sect. 6.4, since the proof in this special case is well-known.

## Infinite covirtually cyclic groups and their finite quotients

### Basics about infinite covirtually cyclic subgroups

A group *V* is called *covirtually cyclic*, if it contains a normal finite subgroup $K \subseteq V$ such that *V*/*K* is cyclic. Equivalently, *V* is either a finite group or there is an extension $1 \rightarrow K \rightarrow V \xrightarrow {\pi _V} C\rightarrow 1$ of groups such that *K* is finite and *C* is infinite cyclic. An infinite covirtually cyclic group is the same as a infinite virtually cyclic group of type I.

Let *V* be a covirtually cyclic subgroup of *G* which is infinite. Then it contains a finite subgroup $K = K_V$ with the property such that any finite subgroup of *G* is contained in *K*. Note that *K* is uniquely determined by this property, is a characteristic and in particular normal subgroup of *V*, and the quotient $C_V:= V/K_V$ is infinite cyclic.

#### Definition 7.1

(*Polarization*) A *polarization of V* is a choice of an element $t \in V$ whose image under the projection $\pi = \pi _V :V \rightarrow C_V$ is a generator.

### Basic definitions and notation

Fix an integer $M \ge 1$. Let *m* be the order of the automorphism $c_t :K \xrightarrow {\cong } K$ given by conjugation with *t*. Then $t^mkt^{-m} = k$ holds for all $k \in K_V$. Hence the infinite cyclic subgroups $\langle t^{Mm}\rangle$ and $\langle t^{m}\rangle$ of *V* are normal and their intersection with $K_V$ is trivial. Define finite groups

$$\begin{aligned} F= &   V/\langle t^{Mm} \rangle ;\\ \widehat{F}= &   V/\langle t^{m} \rangle . \end{aligned}$$

Denote by $\nu :V \rightarrow F$ and $\beta :F \rightarrow \widehat{F}$ the canonical projections. We have the exact sequence $1 \rightarrow \ker (\beta ) \xrightarrow {j} F\xrightarrow {\beta } \widehat{F} \rightarrow 1$.

Note that the following square commutes7.2where the horizontal maps are the obvious isomorphism given by the elements $t \in V$ and $\nu (t) \in F$, and $\beta \circ \nu (t) \in \widehat{F}$, and $p_{Mm} :\mathbb {Z}\rightarrow \mathbb {Z}/Mm$ and $\overline{p}_M :\mathbb {Z}/Mm \rightarrow \mathbb {Z}/m$ are the obvious projections.

### Some properties of subgroups

Consider a subgroup *H* of *F* and a prime *q*. We compute

$$\begin{aligned} [F: H] = \frac{|F|}{|H|} = \frac{M\cdot m\cdot |K_V|}{|H|} = \frac{M\cdot m\cdot |K_V|}{|\beta (H)| \cdot |j^{-1}(H)|} = \frac{M\cdot m\cdot |K_V|\cdot [\widehat{F}: \beta (H)]}{|\widehat{F}| \cdot |j^{-1}(H)|}\\ = \frac{M\cdot m\cdot |K_V|\cdot [\widehat{F}: \beta (H)]}{m\cdot |K_V|\cdot |j^{-1}(H)|} = \frac{M\cdot [\widehat{F}: \beta (H)]}{|j^{-1}(H)|}. \end{aligned}$$

This implies

7.3 $$\begin{aligned} \log _q([F: H])= &   \log _q\left( \frac{M\cdot [\widehat{F}: \beta (H)]}{|j^{-1}(H)|}\right) \end{aligned}$$

7.4 $$\begin{aligned}= &   \log _q(M) + \log _q([\widehat{F}: \beta (H)]) - \log _q(|j^{-1}(H)|) \nonumber \\\ge &   \log _q(M) - \log _q(|j^{-1}(H)|). \end{aligned}$$

Given a prime *p*, a finite group *H* is called *p*-*hyperelementary*, if it can be written as an extension $1 \rightarrow C \rightarrow H \rightarrow P \rightarrow 1$ for a cyclic group *C* and a *p*-group *P*, and and is called *p*-*elementary* if it is isomorphic to $C \times P$ or a cyclic group *C* and a *p*-group *P*. One can always arrange that the order of *C* is prime to *p*.

#### Lemma 7.5

Let *p* be a prime with $p \not = q$. Let $i :K \rightarrow F$ be the injective group homomorphism given by restricting $\nu :V \rightarrow F$ to *K*. Suppose that *q* divides $|i^{-1}(H)|$ and that *H* is a *p*-hyperelementary group. Then

$$\begin{aligned} \log _q([F: H]) \ge \log _q(M). \end{aligned}$$

#### Proof

Note that for $k \in K_V$ and $y \in \ker (\beta )$ we have $\nu (k)\cdot j(y) = j(y) \cdot \nu (k)$. This implies that we get a well-defined group homomorphism

$$\begin{aligned} i^{-1}(H) \times j^{-1}(H) \rightarrow H, \quad (k,y) \mapsto i(k) \cdot j(y). \end{aligned}$$

It is injective by the following calculation for $(k,y) \in (\nu ^{-1}(H) \cap K) \times j^{-1}(H)$ using the fact that $\beta \circ i$ is injective

$$\begin{aligned} e_H = i(k) \cdot j(y)\\ \implies e_{\widehat{F}} = \beta (e_H) = \beta (i(k) \cdot j(y)) = \beta (i(k)) \cdot \beta (j(y)) = \beta (i(k)) \cdot e_{\widehat{F}} = \beta (i(k))\\ \implies \beta \circ i(k) = e_{\widehat{F}} \implies k = e_K \implies k = e_K \;\text {and} \; j(y) = e_H\\ \implies k = e_K \;\text {and} \; y = e_{\ker (\beta )}. \end{aligned}$$

Since *H* is *p*-hyperelementary and $p \not = q$, the *q*-Sylow subgroup of *H* is cyclic. Hence the *q*-Sylow subgroup of $i^{-1}(H) \times j^{-1}(H)$ is cyclic. Since *q* divides $|i^{-1}(H)|$ by assumption, *q* does not divide $|j^{-1}(H)|$ and we get $\log _q(|j^{-1}(H)|) = 0$. Now apply (7.3). $\square$

#### Lemma 7.6

Suppose that *H* is *p*-hyperelementary and that $i^{-1}(H)$ is a *p*-group. Then *H* is *p*-elementary.

#### Proof

By [21, Lemma 3.1] it suffices to show that for every prime *q* with $q\mid |H|$ and $q \not = p$ there exists an epimorphism from *H* onto a non-trivial finite cyclic group of *q*-power order. Let $\alpha :F \rightarrow C_V/(Mm\cdot C_V)$ be the canonical projection, whose kernel is *K*. Since $\alpha$ induces an epimorphism $H \rightarrow \alpha (H)$ onto a finite cyclic group $\alpha (H)$, it suffices to show that the *q*-Sylow subgroup of $\alpha (H)$ is non-trivial for any prime *q* with $q\mid |H|$ and $q \not = p$. Choose an element $h \in H$ and an integer $a \ge 1$ with $h \not = e_H$ and $h^{q^a} = e_H$. It suffices to show that $\alpha (h)$ is not the the unit element. Suppose the contrary. Then we can find $x \in i^{-1}(H)$ with $i(x) = h$. Since $i^{-1}(H)$ is a finite *p*-group, we can choose an integer $b \ge 1$ satisfying $x^{p^b} = e_K$. As $p \not = q$, we can find integers $\lambda , \mu$ with $\lambda q^a + \mu \cdot p^b = 1$. We compute

$$\begin{aligned}i(x) = i(x^{\lambda q^a + \mu p^b}) = (i(x)^{q^a})^{\lambda } \cdot i((x^{p^b})^{\mu }) = (h^{q^a})^{\lambda } \cdot i(e^{\mu }) = e_F \cdot e_F = e_F. \end{aligned}$$

Hence $x = e_K$ which implies $h = e_H$, a contradiction. $\square$

## On Nil-terms for infinite covirtually cyclic subgroups

For the remainder of this section we fix an infinite covirtually cyclic group *V* with a polarization $t \in V$ and a *V*-$\mathbb {Z}$-category $\mathcal {A}$. Let the covariant functor8.1be the functor coming from the functor in (6.1) for $G = V$ and $\nu :V \rightarrow F$ taken from Sect. 7.2

### The basic diagram 8.7

In the sequel we denote for two subgroups $H_0$ and $H_1$ of *V* with $H_0 \subseteq H_1$ by ${{\,\textrm{pr}\,}}:V/H_0 \rightarrow V/H_1$ the *V*-map given by the canonical projection. Given subgroups $H_0$ and $H_1$ of *V* and $v \in V$ in with $v^{-1}H_0v \subseteq H_1$, we denote by $R_{v} :V/H_0 \rightarrow V/H_1$ the *V*-map sending $v'H_0$ to $v'vH_1$. Note that for $v_0,v_1 \in V$ with $v_i^{-1}H_0v_i \subseteq H_1$ for $i = 0,1$ we have $R_{v_0} = R_{v_1}$ if and only if $v_1^{-1}v_0 \in H_1$ holds.

Given $d \in \mathbb {Z}^{\ge 1} = \{ n \in \mathbb {Z}\mid n \ge 1\}$, let $C[d] = d \cdot C \subseteq C$ be the subgroup of index *d* in *C* and *V*[*d*] be the preimage of *C*[*d*] under the projection $\pi :V\rightarrow C$. In particular we get $V[1] = V$.

Let $W\subseteq V$ be a subgroup of *V* of finite index. Then *W* itself is an infinite covirtually cyclic group. Its maximal finite subgroup is $K_W = K_V \cap W$. Let $d = d(W) \in \mathbb {Z}^{\ge 1}$ be the natural number given by the index *d* of $\pi _V(W)$ in the infinite cyclic group *C*. Then $W \subseteq V[d]$ and we have $W = V[d]$ if and only if $K_V \subseteq W$, or, equivalently, $K_W = K_V$ holds.

Fix a polarization $t_W \in W$. Since the following diagram of *V*-spaces commuteswe get from $Z({{\,\textrm{pr}\,}}) :Z(V/K_W) \rightarrow Z(V/W)$ a functor of additive categories

8.2 $$\begin{aligned} U(W,t_W) :Z(V/K_W)_{Z(R_{t_W})}[\mathbb {Z}] \rightarrow Z(V/W). \end{aligned}$$

#### Lemma 8.3

The functor $U(W,t_W)$ is for any choice of polarization $t_W \in W$ an equivalence of additive categories and induces in particular for every $n \in \mathbb {Z}$ an isomorphism

$$\begin{aligned} K_n(U(W,t_W)):K_n(Z(V/K_W)_{Z(R_{t_W})}[\mathbb {Z}]) \rightarrow K_n(Z(V/W)). \end{aligned}$$

#### Proof

This follows from the isomorphism (6.9) and the obvious fact that the canonical map $K_W \rtimes _{c_{t_W}}\mathbb {Z}\rightarrow W$ is an isomorphism. $\square$

We get for $n \in \mathbb {Z}$ from the isomorphism appearing in Lemma 8.3, the twisted Bass-Heller Swan decomposition applied to $\mathcal {A}= Z(V/K_W)$ and $\Phi = Z(R_{t_W})$, see Theorem 4.16, and the projection onto and the inclusion of the first Nil-term maps

$$\begin{aligned} s(W,t_W)_n :\overline{K}_{n-1}({{\,\textrm{Nil}\,}}(Z(V/K_W),Z(R_{t_W})))\rightarrow &   K_n(Z(V/W)); \\ r(W,t_W)_n :K_n(Z(V/W))\rightarrow &   \overline{K}_{n-1}({{\,\textrm{Nil}\,}}(Z(V/K_W),Z(R_{t_W}))), \end{aligned}$$

satisfying $$r(W,t_W)_n \circ s(W,t_W)_n = {{\,\textrm{id}\,}}_{\overline{K}_{n-1}({{\,\textrm{Nil}\,}}(Z(V/K_W),Z(R_{t_W})))}$$.

Fix a polarization $t \in V$ of *V*. The following diagram commutes by Theorem 4.168.4where $V_d$ is the Verschiebungs operator, see (3.2).

Let $W\subseteq V$ be a subgroup of *V* of finite index. Fix a polarization *t* of *V*. Then there is $y \in K = K_V$ with $yt^{d} \in W$. Fix $y = y(W)\in K$ with $yt^{d} \in W$. Then the element $yt^{d}$ is a polarization of *W*. As the composites $V/K_W\xrightarrow {{{\,\textrm{pr}\,}}} V/K \xrightarrow {R_{t^{d}}} V/K$ and $V/K_W \xrightarrow {R_{yt^{d}}} V/K_W \xrightarrow {{{\,\textrm{pr}\,}}} V/K$ agree, $Z({{\,\textrm{pr}\,}}) :Z(V/K_W) \rightarrow Z(V/K)$ induces a map

$$\begin{aligned} \overline{K}_{n-1}(Z({{\,\textrm{pr}\,}})) :\overline{K}_{n-1}({{\,\textrm{Nil}\,}}(Z(V/K_W),Z(R_{yt^{d}}))) \rightarrow \overline{K}_{n-1}({{\,\textrm{Nil}\,}}(Z(V/K),Z(R_{t^{d}}))) \end{aligned}$$

for every $n \in \mathbb {Z}$. One easily easily checks that the following diagram commutes8.5Define

8.6 $$\begin{aligned} \Xi _{n-1}(W,y(W)t^{d(W)}) :\overline{K}_{n-1}({{\,\textrm{Nil}\,}}(Z(V/K_W),Z(R_{y(W)t^{d(W)}}))) \nonumber \\ \rightarrow \overline{K}_{n-1}({{\,\textrm{Nil}\,}}(Z(V/K),Z(R_t))) \end{aligned}$$

to be the composite

$$\begin{aligned} \overline{K}_{n-1}({{\,\textrm{Nil}\,}}(Z(V/K_W),Z(R_{y(W)t^{d(W)}}))) \xrightarrow {{\overline{K}_n(Z({{\,\textrm{pr}\,}}))}} \overline{K}_{n-1}({{\,\textrm{Nil}\,}}(Z/K),Z(R_{t^{d(W)}}))\\ \xrightarrow {\overline{K}_{n-1}(V_d)} \overline{K}_{n-1}({{\,\textrm{Nil}\,}}(Z(V/K),Z(R_t))). \end{aligned}$$

Then we obtain by concatenating (8.4) and (8.5) the commutative diagram8.7

### Improving the induction results

For the remainder of this section consider the setup of Sect. 7.2. In particular we have fixed a natural number *M* and a surjective group homomorphism $\nu :V \rightarrow F$ onto a finite group *F*. Moreover, we fix $n \in \mathbb {Z}$ and let *M*(*Z*, *n*) be the module over the Green functor ${{\,\textrm{Sw}\,}}_F$ associated to *Z* in Sect. 6.1 with respect to the epimorphism $\nu :V \rightarrow F$.

#### Lemma 8.8

Consider any element *z* in $K_n(Z(V/V)) = \mathcal {M}(Z,n)(F/F)$. Let *p* be a prime number. Let $\mathcal {H}_{p}$ be the family of *p*-hyperelementary subgroups of *F*.

Then there are elements $a_H \in \mathbb {Z}_{(p)}$ and $u_H \in {{\,\textrm{Sw}\,}}(H)$ for each $H \in \mathcal {H}_{p}$ such that

$$\begin{aligned} z = \sum _{H \in \mathcal {H}_{p}} a_H \cdot \mathcal {M}(Z,n)_*({{\,\textrm{pr}\,}}_H)\bigl (u_H \cdot \mathcal {M}(Z,n)^*({{\,\textrm{pr}\,}}_H)(z)\bigr ) \end{aligned}$$

holds in $K_n(Z(V/V))_{(p)}$, where ${{\,\textrm{pr}\,}}_H :F/H \rightarrow F/F$ is the projection.

#### Proof

This follows from Lemma 5.9. $\square$

#### Lemma 8.9

Consider an element $z \in \overline{K}_{n-1}({{\,\textrm{Nil}\,}}(Z(V/K_V),Z(R_t)))$ of nilpotence degree $\le D$.

Then for every $H \subseteq F$ satisfying $[F:H] \ge D$ the composite

$$\begin{aligned} \overline{K}_{n-1}({{\,\textrm{Nil}\,}}(Z(V/K_V),Z(R_t))) \xrightarrow {s_n(V,t)} K_n(Z(V/V)) \xrightarrow {\mathcal {M}(Z,n)^*({{\,\textrm{pr}\,}})} K_n(Z(V/\nu ^{-1}(H))) \end{aligned}$$

sends *z* to zero.

#### Proof

Put $d = [F: H] = [V: \nu ^{-1}(H)]$. Then we have $\nu ^{-1}(H) \subseteq V[d]$ and the homomorphism $\mathcal {M}(Z,n)^*({{\,\textrm{pr}\,}}) :K_n(Z(V/V)) \rightarrow K_n(Z(V/\nu ^{-1}(H)))$ is the composite

$$\begin{aligned} K_n(Z(V/V)) \xrightarrow {\mathcal {M}(Z,n)^*({{\,\textrm{pr}\,}})} K_n(Z(V/V[d])) \xrightarrow {\mathcal {M}(Z,n)^*({{\,\textrm{pr}\,}})} K_n(Z(V/\nu ^{-1}(H))). \end{aligned}$$

The following diagram commuteswhere the bijective horizontal arrows have been defined in (8.2). Now the claim follows from Lemma 4.33, since the map $s_n(V,t)$ is by definition the composite

$$\begin{aligned}\overline{K}_{n-1}({{\,\textrm{Nil}\,}}(Z(V/K),Z(R_t))) \xrightarrow {\overline{s}_n} K_n(Z(V/K)_{Z(R_t)}[\mathbb {Z}]) \xrightarrow {K_n(U(V,t))} K_n(Z(V/V)). \end{aligned}$$

$$\square $$

Lemmas 8.8 and 8.9 imply

#### Lemma 8.10

Consider an element *z* in $z \in \overline{K}_{n-1}({{\,\textrm{Nil}\,}}(Z(V/K_V),Z(R_t)))$ of nilpotence degree $\le D$. Let *p* be a prime number. Let $\mathcal {H}_{p}$ be the family of *p*-hyperelementary subgroups of *F*.

Then there are elements $a_H \in \mathbb {Z}_{(p)}$ and $u_H$ for each $H \in \mathcal {H}_{p}$ such that

$$\begin{aligned} s_n(V,t)(z) = \sum _{\begin{array}{c} H \in \mathcal {H}_{p}\\ {[F: H]}< D \end{array}} a_H \cdot \mathcal {M}(Z,n)_*({{\,\textrm{pr}\,}}_H)\bigl (u_H \cdot \mathcal {M}(Z,n)^*({{\,\textrm{pr}\,}}_H) \circ s_n(V,t)(z)\bigr ) \end{aligned}$$

holds in $$K_n(Z(V/V))_{(p)}$$.

Define $\overline{\mathcal {H}_{p}}$ to be the family *p*-hyperelementary subgroups *H* of *F*, for which $i^{-1}(H) = H \cap F \subseteq F$ is a *p*-group.

#### Lemma 8.11

Consider an element *z* in $\overline{K}_{n-1}({{\,\textrm{Nil}\,}}(Z(V/K_V),Z(R_t)))$ of nilpotence degree $\le D$. Let *p* be a prime number. Assume that $\log _q(l) < \log _q(M)$ holds for every natural number *l* with $l < D$ and every prime *q* that satisfies $q \le \max \{D,|\widehat{F}|\}$ and is different from *p*.

Then there are elements $a_H \in \mathbb {Z}_{(p)}$ and $u_H$ for each $H \in \overline{\mathcal {H}_{p}}$ such that

$$\begin{aligned} s_n(V,t)(z) = \sum _{H \in \overline{\mathcal {H}_{p}}} a_H \cdot \mathcal {M}(Z,n)_*({{\,\textrm{pr}\,}}_H)\bigl (u_H \cdot \mathcal {M}(Z,n)^*({{\,\textrm{pr}\,}}_H)\circ s_n(V,t)(z)\bigr ) \end{aligned}$$

holds in $$K_n(Z(V/V))_{(p)}$$.

#### Proof

In view of Lemma 8.10 it suffices to show under the assumptions above that $i^{-1}(H) = H \cap F$ is a *p*-group for $H \in \mathcal {H}_{p}$, provided that $[F:H] < D$ holds. Because of Lemma 7.5 it remains to show $\log _q([F:H]) < \log _q(M)$ for every every $H \in \mathcal {H}_{p}$ and every prime *q* that divides $|i^{-1}(H)| = |H \cap F|$ and is different from *p*, provided that $[F:H] < D$ holds. If *q* satisfies $q \le \max \{D,|\widehat{F}|\}$, then $\log _q([F:H]) < \log _q(M)$ follows from the assumptions. Suppose *q* that satisfies $q > \max \{D,|\widehat{F}|\}$. Then *q* does divide neither [*F* : *H*] nor $\widehat{F}$. Since *q* divides $|H \cap F|$ and hence |*F*| and $|F| = M \cdot |\widehat{F}|$ holds, *q* divides |*M*| and hence $\log _q(M) \ge 1$. As *q* does not divide [*F* : *H*], we have $\log _q([F:H])= 0$ and hence $\log _q([F:H]) < \log _q(M)$. $\square$

#### Notation 8.12

Let  be the set of infinite covirtually cyclic subgroups $W \subseteq V$ such that $K_W = W \cap K_V$ is a *p*-group.

Note that $W \cap K$ is indeed the maximal finite subgroup $K_{W}$ of *W*. For  define the natural number *d*(*W*) to be the index $[C: \pi _V(W)]$, where $\pi _V :V \rightarrow V/K_V = C_V$ is the projection onto the infinite cyclic subgroup $C_V$. For every  choose an element $y(W) \in K_V$ such that $y(W)t^{d(W)}$ is a polarization of *W*. We have defined in (8.6) the map

$$\begin{aligned} \Xi _{n-1}(W,y(W)t^{d(W)}) :\overline{K}_{n-1}\bigl ({{\,\textrm{Nil}\,}}(Z(V/K_{W}),Z(R_{y(W)t^{d(W)}}))\bigr )\\ \rightarrow \overline{K}_{n-1}\bigl ({{\,\textrm{Nil}\,}}(Z(V/K_V),Z(R_t))\bigr ). \end{aligned}$$

The isomorphism type of the abelian group $\overline{K}_{n-1}\bigl ({{\,\textrm{Nil}\,}}(Z(V/K_{W}),Z(R_{y(W)t^{d(W)}}))\bigr )$ and the image of $\Xi _{n-1}(W,y(W)t^{d(W)})$ are independent of the choice of *y*(*W*), since for any other choice $y(W)'$ we have the commutative diagramwith an isomorphisms as vertical arrow.

#### Theorem 8.13

The mapis surjective.

#### Proof

Consider $z \in \overline{K}_{n-1}({{\,\textrm{Nil}\,}}(Z(V/K),Z(R_t)))$. Choose *D* such that the nilpotence degree of *z* is $\le D$. Now choose a natural number *M* such that $\log _q(l) < \log _q(M)$ holds for every natural number *l* with $l < D$ and every prime *q* that satisfies $q \le \max \{D,|\widehat{F}|\}$ and is different from *p*. From Lemma 8.11 we get elements $a_H \in \mathbb {Z}_{(p)}$ and $u_H$ for each $H \in \overline{\mathcal {H}_{p}}$ such that

8.14 $$\begin{aligned} s(V,t)_n(z) = \sum _{H \in \overline{\mathcal {H}_{p}}} a_H \cdot \mathcal {M}(Z,n)_*({{\,\textrm{pr}\,}}_H)\bigl (u_H \cdot \mathcal {M}(Z,n)^*({{\,\textrm{pr}\,}}_H)\circ s_n(V,t)(z)\bigr )\nonumber \\ \end{aligned}$$

holds in $K_n(Z(V/V))_{(p)}$. Define for $H \in \overline{\mathcal {H}_p}$ the subgroup $W_H$ of *V* by $W_H:= \nu ^{-1}(H)$. For $H \in \overline{\mathcal {H}_p}$ the following diagram8.15commutes because of the commutative diagram (8.7). Define the element in $z_H \in \overline{K}_{n-1}\bigl ({{\,\textrm{Nil}\,}}(Z(V/K_{W}),Z(R_{y_{W}t^{d(W)}}))\bigr )_{(p)}$ by

8.16 $$\begin{aligned} z_H = r(W_H,y_{W_H}t^{d(W_H)})_n\circ \bigl (u_H \cdot \mathcal {M}(Z,n)^*({{\,\textrm{pr}\,}}_H)\circ s(V,t)_n(z)\bigr ). \end{aligned}$$

Recall that we have $r(V,t)_n \circ s(V,T)_n = {{\,\textrm{id}\,}}$. Now we compute

$$\begin{aligned} z  &   = r(V,t)_n \circ s(V,T)_n(z)\\  &   {\mathop {=}\limits ^{(8.14)}} r(V,T)_n \circ \biggl (\sum _{H \in \overline{\mathcal {H}_{p}}} a_H \cdot \mathcal {M}(Z,n)_*({{\,\textrm{pr}\,}}_H)\bigl (u_H \cdot \mathcal {M}(Z,n)^*({{\,\textrm{pr}\,}}_H)\circ s(V,t)_n(z)\bigr )\biggr )\\  &   = \sum _{H \in \overline{\mathcal {H}_{p}}} a_H \cdot r(V,T)_n \circ \mathcal {M}(Z,n)_*({{\,\textrm{pr}\,}}_H)\bigl (u_H \cdot \mathcal {M}(Z,n)^*({{\,\textrm{pr}\,}}_H) \circ s(V,t)_n(z)\bigr )\\  &   {\mathop {=}\limits ^{(8.15)}} \sum _{H \in \overline{\mathcal {H}_{p}}} a_H \cdot \Xi _{n-1}(W_H,y_{W_H}t^{d(W_H)}) \circ r(W_H,y(W_H)t^{d(W_H)})_n\\  &   \quad \circ \bigl (u_H \cdot \mathcal {M}(Z,n)^*({{\,\textrm{pr}\,}}_H)\circ s(V,t)_n(z)\bigr )\\  &   {\mathop {=}\limits ^{(8.16)}} \sum _{H \in \overline{\mathcal {H}_{p}}} a_H \cdot \Xi _{n-1}(W_H,y(W_H)t^{d(W_H)})(z_H). \end{aligned}$$

Note that for $H \in \overline{\mathcal {H}_p}$ the subgroup $W_H:= \nu ^{-1}(H)$ of *V* belongs to  introduced in Notation 8.12. This finishes the proof of Theorem 8.13. $\square$

#### Corollary 8.17

If $\overline{K}_{n-1}\bigl ({{\,\textrm{Nil}\,}}(Z(V/K_{W}),Z(R_{y_{W}t^{d(W)}}))\bigr )_{(p)} = 0$ holds for every , then we get

$$\begin{aligned} \overline{K}_{n-1}\bigl ({{\,\textrm{Nil}\,}}(Z(V/K),Z(R_t))\bigr )_{(p)} = 0. \end{aligned}$$

## Proof of Theorems 1.2 and 1.5 for additive categories

Let *G* be a group and $\Lambda$ be a commutative ring. Let $\mathcal {A}$ be an additive *G*-$\Lambda$-category. We have defined $\mathcal {P}(G,\Lambda )$ in Notation 1.1.

### Theorem 9.1

Suppose that the additive category $\mathcal {A}$ is regular in the sense of [4, Definition 6.2]. Then the canonical map

$$\begin{aligned} H_n^G(E_{{{{\mathcal {F}}}\textrm{in}}}(G);\textbf{K}_{\mathcal {A}}) \rightarrow H_n^G(E_{{{{\mathcal {V}}}\textrm{cyc}}}(G);\textbf{K}_{\mathcal {A}}) \end{aligned}$$

is a $\mathcal {P}(G,\Lambda )$-isomorphism for all $n \in \mathbb {Z}$.

### Proof

The relative assembly map

$$\begin{aligned} H_n^G(E_{{\mathcal {C}\textrm{vcy}}}(G);\textbf{K}_{\mathcal {A}}) \rightarrow H_n^G(E_{{{{\mathcal {V}}}\textrm{cyc}}}(G);\textbf{K}_{\mathcal {A}}) \end{aligned}$$

is an isomorphism for all $n \in \mathbb {Z}$ by [16, Remark 1.6], see also [25, Theorem 13.44]. Hence it suffices to show that the relative assembly map

$$\begin{aligned} H_n^G(E_{{{{\mathcal {F}}}\textrm{in}}}(G);\textbf{K}_{\mathcal {A}}) \rightarrow H_n^G(E_{{\mathcal {C}\textrm{vcy}}}(G);\textbf{K}_{\mathcal {A}}) \end{aligned}$$

is a $\mathcal {P}(G,\Lambda )$-isomorphism for all $n \in \mathbb {Z}$. By the Transitivity Principle appearing in [2, Theorem 3.3], see also [25, Theorem 15.12], we can assume without loss of generality that *G* itself is an infinite covirtually cyclic group *V*. Hence we have to show for any covirtually cyclic group *V* that the assembly map

9.2 $$\begin{aligned} H_n^V(E_{{{{\mathcal {F}}}\textrm{in}}}(V);\textbf{K}_{\mathcal {A}}) \rightarrow H_n^V(E_{{\mathcal {C}\textrm{vcy}}}(V);\textbf{K}_{\mathcal {A}}) = H_n^V(V/V;\textbf{K}_{\mathcal {A}}) = \pi _n(\textbf{K}_{\mathcal {A}}(V)) \nonumber \\ \end{aligned}$$

is a $\mathcal {P}(G,\Lambda )$-isomorphism, where we view $\mathcal {A}$ as a *V*-$\Lambda$-category by restriction from *G* to *V*. Write $V = K \rtimes _{c_t} \mathbb {Z}$. The twisted Bass-Heller-Swan isomorphism of (4.5) applied to $V = K \rtimes _{c_t} \mathbb {Z}$ yields an isomorphism

$$\begin{aligned} \pi _n(T_{\textbf{K}(c_t)}) \times \overline{K}_{n-1}\bigl ({{\,\textrm{Nil}\,}}(Z_{\mathcal {A}}(V/K),Z(R_t))\bigr ) \times \overline{K}_{n-1}\bigl ({{\,\textrm{Nil}\,}}(Z_{\mathcal {A}}(V/K),Z(R_t))\bigr ) \\ \xrightarrow {\cong } \pi _n(\textbf{K}_{\mathcal {A}}(V)). \end{aligned}$$

There is an identification

$$\begin{aligned} \pi _n(T_{\textbf{K}(c_t)}) \xrightarrow {\cong } H_n^V(E_{{{{\mathcal {F}}}\textrm{in}}}(V);\textbf{K}_{\mathcal {A}}) \end{aligned}$$

coming from the fact that a model for $E_{{{{\mathcal {F}}}\textrm{in}}}(V)$ is $\mathbb {R}$ with respect to the *V*-action given by $v \cdot r = {{\,\textrm{pr}\,}}(v) + r$ for $r \in \mathbb {R}$ and the canonical projection ${{\,\textrm{pr}\,}}:V \rightarrow \mathbb {Z}$. Under these identifications the map (9.2) becomes the obvious inclusion. Hence map (9.2) is a $\mathcal {P}(G,\Lambda )$-isomorphism if we can show

$$\begin{aligned} \mathbb {Z}[\mathcal {P}(G,\Lambda )^{-1}] \otimes _{\mathbb {Z}} \overline{K}_{n-1}\bigl ({{\,\textrm{Nil}\,}}(Z_{\mathcal {A}}(V/K),Z(R_t))\bigr ) = 0 \end{aligned}$$

for every $n \in \mathbb {Z}$. Because of Corollary 8.17 it suffices to show that for any $p \not \in \mathcal {P}(G,\Lambda )$ and any  we have $\overline{K}_{n-1}\bigl ({{\,\textrm{Nil}\,}}(Z_{\mathcal {A}}(G/K_{W}),Z(R_{y_{W}t^{d(W)}}))\bigr )= 0$.

Because of [4, Lemma 7.7 and Theorem 8.1] it suffices to show that the additive category $Z_{\mathcal {A}}(G/K_{W})$ is regular. Since it is equivalent to $\mathcal {A}|_{K_W}[K_W]$ and $K_W$ is a *p*-group, it suffices to show for any *p*-subgroup *P* of *V* that $\mathcal {A}|_P[P]$ is regular. If the *p*-subgroup *P* is trivial, then $\mathcal {A}|_P[P] = \mathcal {A}$ and hence by assumption regular. If *P* is non-trivial, then by definition of $\mathcal {P}(V,\Lambda )$ the prime *p* is invertible in $\Lambda$. We leave it to the reader to check that then $\mathcal {A}|_P[P]$ is regular, as $\mathcal {A}$ is regular. It is not hard to extend the proof for rings in [7, Lemma 7.4 (2)] to additive categories. This finishes the proof of Theorem 9.1. $\square$

### Corollary 9.3

Suppose *G* satisfies the Full Farrell–Jones Conjecture. Assume that the additive $\Lambda$-category $\mathcal {A}$ is regular in the sense of [4, Definition 6.2] and that the order of any finite subgroup of *G* is invertible in $\Lambda$.

Then the canonical mapis an isomorphism and

$$\begin{aligned} K_n(\mathcal {A}[G/G]) = 0 \quad \text {for} \;n \le -1. \end{aligned}$$

where $\mathcal {A}[G/G]$ has been defined in Sect. 6.1.

### Proof

This follows from Theorem 9.1 using the equivariant Atiyah–Hirzebruch spectra sequence and the fact that $\mathcal {A}(G/H)$ is equivalent to the regular additive category $\mathcal {A}|_H[H]$ for finite $H \subseteq G$, which implies $K_i(\mathcal {A}(G/H)) = 0$ for $i \le -1$ and $|H| < \infty$, see for instance [25, Proof of Proposition 13.48 (iv)], [8, Section 4]. $\square$

## Nil-terms as modules over the ring of big Witt vectors

We extend the result of Weibel [41, Corollary 3.2], which is based on work by Stienstra [35], that for a ring *R* of characteristic *N* for some natural number *N* we have $N\!K_n(RG)[1/N] = \{0\}$ for every group *G* and every $n \in \mathbb {Z}$, to additive categories allowing twisting by an automorphism.

### Review of the ring of big Witt vectors

Let $\Lambda$ be a commutative ring. Let $W(\Lambda )$ be the commutative ring of big Witt vectors. The underlying abelian group is the multiplicative group $1 + t\Lambda [[t]]$ of formal power series $1 + \lambda _1t + \lambda _2t^2 + \cdots$ in *t* with coefficients in $\Lambda$ and leading term 1. We do not give the details of the multiplicative structure $*$ but at least mention that it is the unique continuous functional with the property that $(1 -\lambda t) *(1-\mu t) = (1- \lambda \mu t)$ holds for $\lambda , \mu \in \Lambda$. We mention that it satisfies for $m,n \in \mathbb {Z}^{\ge 1}$ and $\lambda , \mu \in \Lambda$

10.1 $$\begin{aligned} (1- \lambda t^m) *(1- \mu t^n) = (1 - \lambda ^{n/d}\mu ^{m/d}t^{mn/d})^d, \end{aligned}$$

where *d* is greatest common divisor of *m* and *n*. The unit for the addition is 1, whereas the unit for the multiplication is $(1-t)$.

For $N \in \mathbb {Z}^{\ge 1}$ the subgroup $I_N = 1 + t^N\Lambda [[t]]$ is actually an ideal in $W(\Lambda )$. We have $W(\Lambda ) = I_1 \supset I_2 \supset I_3 \supset \cdots$ and $\bigcap _{N \ge 1} I_N = \{1\}$. Thus we obtain the so called *t*-adic topology on $W(\Lambda )$. Then $W(\Lambda )$ is separated and complete in this topology. The quotient rings $W_N(\Lambda ) = W(\Lambda )/I_{N+1}$ are called the rings of *N*-truncated Witt vectors. For more information we refer to [13, Section I.1].

### Endomorphisms rings

Let ${{\,\textrm{End}\,}}(\Lambda )$ be the exact category of endomorphism of finitely generated projective $\Lambda$-modules. Objects are pairs (*P*, *f*) consisting of a finitely generated projective $\Lambda$-module *P* together with a $\Lambda$-endomorphism $f :P \rightarrow P$. A morphisms $g :(P_0,f_0) \rightarrow (P_1,f_1)$ is a $\Lambda$-homomorphism $g :P_0 \rightarrow P_1$ satisfying $f_1 \circ g = g \circ f_0$. We have the inclusion  sending a finitely generated projective $\Lambda$-module *P*, to (*P*, 0). It has a retraction  sending (*P*, *f*) to *P*. Let $K_0({{\,\textrm{End}\,}}(\Lambda ))$ and  be the projective class groups associated to the exact categories ${{\,\textrm{End}\,}}(\Lambda )$ and . Define the reduced projective class group10.2Note that then we get from *i* and *r* a natural isomorphism

10.3 $$\begin{aligned} K_0({{\,\textrm{End}\,}}(\Lambda )) \xrightarrow {\cong } K_0(\Lambda ) \oplus \overline{K}_0({{\,\textrm{End}\,}}(\Lambda )). \end{aligned}$$

Note that the tensor product over $\Lambda$ induces the structure of a commutative ring on $K_0({{\,\textrm{End}\,}}(\Lambda ))$. Since the image of $K_0(i)$ is an ideal, the quotient $\overline{K}_0({{\,\textrm{End}\,}}(\Lambda ))$ inherits the structure of a commutative ring.

There is a well-defined map $\eta :\overline{K}_0({{\,\textrm{End}\,}}(\Lambda )) \rightarrow W(\Lambda )$, which sends the class of (*P*, *f*) for an endomorphisms $f :P \rightarrow P$ of a finitely generated projective $\Lambda$-module *P* to its characteristic polynomial $\det _{\Lambda }(1 -tf)$. The next result is due to Almkvist [1].

#### Theorem 10.4

We obtain a well-defined injective ring homomorphism

$$\begin{aligned} \eta :\overline{K}_0({{\,\textrm{End}\,}}(\Lambda )) \rightarrow W(\Lambda ) \end{aligned}$$

whose image consists of all rational functions, i.e., quotients $x/x'$ of polynomials $x,x'$ in $1 + t \Lambda [t] \subseteq 1 + t \Lambda [[t]]$.

The *t*-adic topology on $\overline{K}_0({{\,\textrm{End}\,}}(\Lambda ))$ is given by the filtration $\overline{K}_0({{\,\textrm{End}\,}}(A)) = \eta ^{-1}(I_1) \supset \eta ^{-1}(I_2) \supset \eta ^{-1}(I_3) \supset \cdots$. Let $\overline{K}_0({{\,\textrm{End}\,}}(A))^{\widehat{\phantom{a}}}$ be the completion of $\overline{K}_0({{\,\textrm{End}\,}}(A))$ with respect to the *t*-adic topology. Since $W(\Lambda )$ is separated and complete in the *t*-adic topology and the image of $\eta$ is dense in $W(\Lambda )$, we conclude from Theorem 10.4 that $\eta$ induces an isomorphism of topological rings

10.5 $$\begin{aligned} \widehat{\eta } :\overline{K}_0({{\,\textrm{End}\,}}(A))^{\widehat{\phantom{a}}}\xrightarrow {\cong } W(\Lambda ). \end{aligned}$$

### The action on Nil-groups

Let $\mathcal {A}$ be an additive $\Lambda$-category, i.e., a small category enriched over the category of $\Lambda$-modules coming with a direct sum $\oplus$ compatible with the $\Lambda$-module structures. Let $\Phi :\mathcal {A}\xrightarrow {\cong } \mathcal {A}$ be an automorphism of additive $\Lambda$-categories of $\mathcal {A}$. Denote by ${{\,\textrm{Nil}\,}}(\mathcal {A};\Phi )$ the associated Nil-category, which inherits the structure of an exact category. Recall that then the *K*-groups $K_s({{\,\textrm{Nil}\,}}(\mathcal {A};\Phi ))$ and $\overline{K}_s({{\,\textrm{Nil}\,}}(\mathcal {A};\Phi ))$ are defined for $s \in \mathbb {Z}$.

Let $\underline{\Lambda }_{\oplus }$ be the additive $\Lambda$-category obtained from the obvious $\Lambda$-category having precisely one object by adding finite sums. More precisely, for every $m \in \mathbb {Z}^{\ge }$ we have the object [*m*] in $\underline{\Lambda }$. A morphism $C :[m] \rightarrow [n]$ for $m,n \ge 1$ is a (*m*, *n*)-matrix $C = (c_{i,j})$ over $\Lambda$. The object [0] is declared to be the zero object. Composition is given by matrix multiplication, whereas the direct sum is given on objects by assigning to two object [*m*] and [*n*] the object $[m+n]$ and on morphisms by the block sum of matrices.

We define a bilinear functor of additive $\Lambda$-categories

10.6 $$\begin{aligned} F :\underline{\Lambda }_{\oplus } \times \mathcal {A}\rightarrow \mathcal {A} \end{aligned}$$

as follows. On objects it is defined by sending ([*m*], *A*) to $\bigoplus _{i = 1}^m A$. Consider morphisms $C :[m] \rightarrow [n]$ and $f :A \rightarrow B$ in $\underline{\Lambda }$ and $\mathcal {A}$. Define the morphism

$$\begin{aligned} (C \otimes f) = (C \oplus f)_{i,j} :C([m],A) = \bigoplus _{i = 1}^m A \rightarrow C([n],B) =\bigoplus _{j= 1}^n B \end{aligned}$$

by $(C \oplus f)_{i,j} = c_{i,j} \cdot f :A \rightarrow B$ for $(i,j) \in \{1, \ldots , m \} \times \{1, \ldots , n \}$. If we fix an object ([*n*], *C*) in ${{\,\textrm{End}\,}}(\underline{\Lambda })$, we get a functor of exact categories $F_{([n],C)} :{{\,\textrm{Nil}\,}}(\mathcal {A};\Phi ) \rightarrow {{\,\textrm{Nil}\,}}(\mathcal {A};\Phi )$ by sending an object $(A,\varphi )$ that is given by the nilpotent endomorphism $\varphi :\Phi (A) \rightarrow A$ to the object that is given by the nilpotent endomorphism defined by the composite

$$\begin{aligned} \Phi \left( \bigoplus _{i=1}^n A\right) \xrightarrow {\sigma (n,A)^{-1}} C([n],\Phi (A)) = \bigoplus _{i=1}^n \Phi (A) \xrightarrow {F(C,\varphi )} C([n],A) = \bigoplus _{i=1}^n A \end{aligned}$$

for the canonical isomorphism $\sigma (n,A) :\bigoplus _{i=1}^n \Phi (A) \xrightarrow {\cong } \Phi \left( \bigoplus _{i=1}^n A\right)$. It induces a homomorphism $\overline{K}_n(F_{([n],C)}) :\overline{K}_n({{\,\textrm{Nil}\,}}(\mathcal {A};\Phi )) \rightarrow \overline{K}_n({{\,\textrm{Nil}\,}}(\mathcal {A};\Phi ))$ for every $n \in \mathbb {Z}$. Now one easily checks that the collection of these homomorphisms defines a bilinear pairing of abelian groups for every $s \in \mathbb {Z}$

10.7 $$\begin{aligned} T_s :\overline{K}_0({{\,\textrm{End}\,}}(\Lambda )) \times \overline{K}_s({{\,\textrm{Nil}\,}}(\mathcal {A};\Phi )) \rightarrow \overline{K}_s({{\,\textrm{Nil}\,}}(\mathcal {A};\Phi )). \end{aligned}$$

#### Lemma 10.8

The pairing $T_s$ of (10.7) extends uniquely to continuous pairing

$$\begin{aligned} \widehat{T}_s :W(\Lambda ) \times \overline{K}_s({{\,\textrm{Nil}\,}}(\mathcal {A};\Phi )) \rightarrow \overline{K}_s({{\,\textrm{Nil}\,}}(\mathcal {A};\Phi )) \end{aligned}$$

for $s \in \mathbb {Z}$, where we equip $\overline{K}_s({{\,\textrm{Nil}\,}}(\mathcal {A};\Phi ))$ with the discrete topology and $W(\Lambda )$ with the *t*-adic topology.

Moreover, this pairing turns $\overline{K}_s({{\,\textrm{Nil}\,}}(\mathcal {A};\Phi ))$ into a $W(\Lambda )$-module.

#### Proof

Because of the isomorphism of topological rings (10.5), the existence of the extension $\widehat{T}_s$ follows if we can show for every element $y \in \overline{K}_s({{\,\textrm{Nil}\,}}(\mathcal {A};\Phi ))$ that there exists an integer *N* such that for every $x \in \eta ^{-1}(I_N)$ we have $T_s(x,y) = y$. This is done as follows.

In the first step we reduce the claim to the special case $s \ge 1$.

From [27, Definition 2.1, Theorem 3.4 and Lemma 6.5] applied to the functor sending $(\mathcal {A}, \Phi )$ to the connective *K*-theory spectrum $\textbf{K}({{\,\textrm{Nil}\,}}(\mathcal {A},\Phi ))$, we get a natural (untwisted) Bass–Heller–Swan isomorphisms for $s \in \mathbb {Z}$

$$\begin{aligned} K_{s-1}({{\,\textrm{Nil}\,}}(\mathcal {A},\Phi )) \oplus K_{s}({{\,\textrm{Nil}\,}}(\mathcal {A},\Phi )) \oplus N\!K_{s}({{\,\textrm{Nil}\,}}(\mathcal {A},\Phi )) \oplus N\!K_{s}({{\,\textrm{Nil}\,}}(\mathcal {A},\Phi ))\\ \xrightarrow {\cong } K_{s}({{\,\textrm{Nil}\,}}(\mathcal {A}[\mathbb {Z}],\Phi [\mathbb {Z}])). \end{aligned}$$

Restricting this isomorphism to $K_{s-1}({{\,\textrm{Nil}\,}}(\mathcal {A},\Phi ))$ yields a (split) injective homomorphisms $\sigma _s :K_{s-1}({{\,\textrm{Nil}\,}}(\mathcal {A},\Phi )) \rightarrow K_{s}({{\,\textrm{Nil}\,}}(\mathcal {A}[\mathbb {Z}],\Phi [\mathbb {Z}]))$, natural in $(\mathcal {A}, \Phi )$. It induces a (split) injective homomorphism $\overline{\sigma }_s :\overline{K}_{s-1}({{\,\textrm{Nil}\,}}(\mathcal {A},\Phi )) \rightarrow \overline{K}_{s}({{\,\textrm{Nil}\,}}(\mathcal {A}[\mathbb {Z}],\Phi [\mathbb {Z}]))$, natural in $(\mathcal {A}, \Phi )$, since we also have the (untwisted) Bass-Heller-Swan isomorphism

$$\begin{aligned} K_{s-1}(\mathcal {A}) \oplus K_{s}(\mathcal {A}) \oplus N\!K_{s}(\mathcal {A}) \oplus N\!K_{s}(\mathcal {A}) \xrightarrow {\cong } K_{s}(\mathcal {A}[\mathbb {Z}]). \end{aligned}$$

Now we obtain for every $s \in \mathbb {Z}$ a commutative diagramSince for every integer *s* we can find a natural number *k* with $s + k \ge 1$ and we can iterate the construction of the diagram above *k*-times, it suffices to show for every element $y \in \overline{K}_s({{\,\textrm{Nil}\,}}(\mathcal {A};\Phi ))$ for $s \ge 1$ that there exists an integer *N* such that for every $x \in \eta ^{-1}(I_N)$ we have $T_s(x,y) = y$. Moreover, we can work for the rest of the proof with the connective *K*-theory spectrum of Waldhausen categories.

Let ${{\,\textrm{Nil}\,}}(\mathcal {A};\Phi )_N$ be the full exact subcategory of ${{\,\textrm{Nil}\,}}(\mathcal {A};\Phi )$ consisting of those objects $(A,\varphi )$, whose nilpotence degree is less or equal to *N*, i.e., $\varphi ^{(N)} :\Phi ^N(A) \rightarrow A$ is trivial. For every natural number *N* the construction of the pairing $T_s$ of (10.7) yields also a pairing

10.9 $$\begin{aligned} T[N]_s :\overline{K}_0({{\,\textrm{End}\,}}(\Lambda )) \times \overline{K}_s({{\,\textrm{Nil}\,}}(\mathcal {A};\Phi )_N) \rightarrow \overline{K}_s({{\,\textrm{Nil}\,}}(\mathcal {A};\Phi )). \end{aligned}$$

These pairings $T[N]_s$ and $T_s$ are compatible with the map $\overline{K}_s({{\,\textrm{Nil}\,}}(\mathcal {A};\Phi )_N) \rightarrow \overline{K}_s({{\,\textrm{Nil}\,}}(\mathcal {A};\Phi ))$ coming from the obvious inclusions of full subcategories. In view of the isomorphism (4.31), it suffices to show for every natural number *N* and $s \ge 1$ that $T[N]_s(x,y) = 0$ holds for $x \in \eta ^{-1}(I_N)$ and $y \in \overline{K}_n({{\,\textrm{Nil}\,}}(\mathcal {A};\Phi )_N)$.

For a natural number $n \ge 1$ and $\lambda _1, \lambda _2, \dots , \lambda _n$ in $\Lambda$, define the automorphism $C_n(\lambda _1, \lambda _2, \ldots , \lambda _r;\varphi ) :[n] \rightarrow [n]$ in ${{\,\textrm{End}\,}}(\Lambda )$ by the (*n*, *n*)-matrix over $\Lambda$

$$\begin{aligned} C_n(\lambda _1, \lambda _2, \ldots , \lambda _n) = \begin{pmatrix} 0 &  0 &  0 &  \cdots &  0 &  0 &  \lambda _n \\ 1 &  0 &  0 &  \cdots &  0 &  0 &  \lambda _{n-1} \\ 0 &  1 &  0 &  \cdots &  0 &  0 &  \lambda _{n-2} \\ 0 &  0 &  1 &  \cdots &  0 &  0 &  \lambda _{n-2} \\ 0 &  0 &  0 &  \cdots &  0 &  0 &  \lambda _{n-3} \\ \vdots &  \vdots &  \vdots &  \ddots &  \vdots &  \vdots &  \vdots \\ 0 &  0 &  0 &  \cdots &  0 &  0 &  \lambda _3 \\ 0 &  0 &  0 &  \cdots &  1 &  0 &  \lambda _2 \\ 0 &  0 &  0 &  \cdots &  0 &  1 &  \lambda _1 \end{pmatrix}. \end{aligned}$$

Then $\eta \bigl ([C_n(-\lambda _1, -\lambda _2, \ldots -\lambda _n)]\bigr ) = 1 +\lambda _1 t + \lambda \cdot t^2 + \cdots + \lambda _n \cdot t^n$. Because of Theorem 10.4 it suffices to prove $T[N]_n([C_n(\lambda _1, \lambda _2, \ldots \lambda _n)],y) = 0$ for every $s,N \ge 1$, elements $\lambda _1, \lambda _2, \ldots , \lambda _n$ in $\Lambda$ and $y \in \overline{K}_s({{\,\textrm{Nil}\,}}(\mathcal {A};\Phi )_N)$, provided that $\lambda _i = 0$ holds for $i = 1,2 \ldots , N-1$.

Fix $s,N \ge 1$ and $\lambda _1, \lambda _2, \ldots , \lambda _n$ in $\Lambda$ such that $\lambda _i = 0$ holds for $i = 1,2 \ldots , N-1$. In the sequel we use the notation of [29, Section 8]. We have defined the functor of Walhausen categoriesin (4.24). It induces an isomorphism10.10for $s \ge 1$, see [29, Theorem 8.1]. Note that in [29, Theorem 8.1] the category $\mathcal {A}$ is assumed to be idempotent complete but this does not matter, since the maps $K_s({{\,\textrm{Nil}\,}}(\mathcal {A},\Phi )) \rightarrow K_s({{\,\textrm{Nil}\,}}({{\,\textrm{Idem}\,}}(\mathcal {A}),{{\,\textrm{Idem}\,}}(\Phi )))$ and  induced by the inclusions are isomorphisms for $s \ge 1$ by the usual cofinality argument, see for instance [27, page 225], where ${{\,\textrm{Idem}\,}}$ denotes idempotent completion.

Given a natural number $n \ge 1$, elements $\lambda _1, \lambda _1, \ldots , \lambda _n \in \Lambda$, and a nilpotent endomorphism $\varphi :\Phi (A) \rightarrow A$ in $\mathcal {A}$, define the morphism in $\mathcal {A}_{\Phi }[t^{-1}]$

$$\begin{aligned} U_{\lambda _1,\lambda _2, \ldots , \lambda _n}(\varphi ) :\Phi (A)^n \rightarrow A^n \end{aligned}$$

by the (*n*, *n*)-matrix

$$\begin{aligned} \begin{pmatrix} {{\,\textrm{id}\,}}_{A} \cdot t^{-1} &  0 &  0 &  \cdots &  0 &  0 &  0 &  -(\lambda _n \cdot \varphi ) \cdot t^0 \\ -\varphi \cdot t^0 &  {{\,\textrm{id}\,}}_{A} \cdot t^{-1} &  0 &  \cdots &  0 &  0 &  0 &  -(\lambda _{n-1} \cdot \varphi ) \cdot t^0 \\ 0 &  -\varphi \cdot t^0 &  {{\,\textrm{id}\,}}_{A} \cdot t^{-1} &  \cdots &  0 &  0 &  0 &  -(\lambda _{n-2} \cdot \varphi ) \cdot t^0 \\ \vdots &  \vdots &  \vdots &  \ddots & \vdots &  \vdots &  \vdots &  \vdots \\ 0 &  0 &  0 & \cdots &  -\varphi \cdot t^0 &  {{\,\textrm{id}\,}}_{A} \cdot t^{-1} &  0 &  -(\lambda _{3} \cdot \varphi ) \cdot t^0 \\ 0 &  0 &  0 & \cdots &  0 &  -\varphi \cdot t^0 &  {{\,\textrm{id}\,}}_{A} \cdot t^{-1} &  - (\lambda _{2} \cdot \varphi ) \cdot t^0 \\ 0 &  0 &  0 & \cdots &  0 &  0 &  - \varphi \cdot t^0 &  {{\,\textrm{id}\,}}_{A} \cdot t^{-1} -(\lambda _{1} \cdot \varphi ) \cdot t^0 \end{pmatrix}. \end{aligned}$$

Thus we obtain a functor of Waldhausen categorieswhich induces a homomorphismThis map can easily be identified with the map sending $y \in K_s({{\,\textrm{Nil}\,}}(\mathcal {A},\Phi ))$ to the image of $T_s([C_n(\lambda _1, \lambda _2, \ldots \lambda _n)],y) = 0$ under the injective homomorphism (10.10). Hence it suffices to show that the image of the compositeis contained in the image ofwhere *J* is the obvious inclusion, and *I* sends an object *A* to (*A*, 0).

Given a natural number $n \ge 1$ and a nilpotent endomorphism $\varphi :\Phi (A) \rightarrow A$ in $\mathcal {A}$, define the automorphism in $\mathcal {A}_{\Phi }[t,t^{-1}]$

$$\begin{aligned} E_n(\varphi ) :A^n \xrightarrow {\cong } A^n \end{aligned}$$

by the (*n*, *n*)-matrix

$$\begin{aligned} \begin{pmatrix} {{\,\textrm{id}\,}}_{\Phi (A)} \cdot t^0&  0 &  0 &  \cdots &  0 &  0 &  0 &  0 \\ \varphi \cdot t &  {{\,\textrm{id}\,}}_{\Phi (A)} \cdot t^0&  0 &  \cdots &  0 &  0 &  0 &  0 \\ (\varphi \cdot t)^2 &  \varphi \cdot t &  {{\,\textrm{id}\,}}_{\Phi (A)} \cdot t^0 & \cdots &  0 &  0 &  0 &  0 \\ \vdots &  \vdots &  \vdots &  \ddots & \vdots &  \vdots &  \vdots &  \vdots \\ (\varphi \cdot t)^{n-3} &  (\varphi \cdot t)^{n-4} &  (\varphi \cdot t)^{n-5} & \cdots &  \varphi \cdot t &  {{\,\textrm{id}\,}}_{\Phi (A)} \cdot t^0&  0 &  0 \\ (\varphi \cdot t)^{n-2} &  (\varphi \cdot t)^{n-3} &  (\varphi \cdot t)^{n-4} & \cdots &  (\varphi \cdot t)^2 &  \varphi \cdot t &  {{\,\textrm{id}\,}}_{\Phi (A)} \cdot t^0 &  0 \\ (\varphi \cdot t)^{n-1} &  (\varphi \cdot t)^{n-2} &  (\varphi \cdot t)^{n-3} & \cdots &  (\varphi \cdot t)^3 &  (\varphi \cdot t)^2 &  \varphi \cdot t &  {{\,\textrm{id}\,}}_{\Phi (A)} \cdot t^0 \end{pmatrix}. \end{aligned}$$

Then we get in $\mathcal {A}_{\Phi }[t,t^{-1}]$ that the composite $F_n(\varphi ):= E_n(\varphi ) \circ U_{\lambda _1,\lambda _2, \ldots , \lambda _n}(\varphi ) :\Phi (A)^n \rightarrow A^n$ is given by the matrix

$$\begin{aligned} \begin{pmatrix} {{\,\textrm{id}\,}}_{A} \cdot t^{-1} &  0 &  0 & \cdots &  0 &  0 &  0 &  v_1(\varphi ) \\ 0 &  {{\,\textrm{id}\,}}_{A} \cdot t^{-1} &  0 &  \cdots &  0 &  0 &  0 &  v_2(\varphi ) \\ 0 & 0&  {{\,\textrm{id}\,}}_{A} \cdot t^{-1} &  \cdots &  0 &  0 &  0 &  v_3(\varphi ) \\ \vdots &  \vdots &  \vdots &  \ddots & \vdots &  \vdots &  \vdots &  \vdots \\ 0 &  0 &  0 &  \cdots &  0 &  {{\,\textrm{id}\,}}_{A} \cdot t^{-1} &  0 &  v_{n-2}(\varphi ) \\ 0 &  0 &  0 &  \cdots &  0 &  0 &  {{\,\textrm{id}\,}}_{A} \cdot t^{-1} &  v_{n-1}(\varphi ) \\ 0 &  0 &  0 &  \cdots &  0 &  0 &  0 &  {{\,\textrm{id}\,}}_{A} \cdot t^{-1} -v_n(\varphi ) \end{pmatrix} \end{aligned}$$

where we define for $k = 1,2,, \ldots n$

$$\begin{aligned} v_n(\varphi ) = \sum _{i = 0}^{k-1} (\varphi \cdot t)^{i} \circ \bigl ((\lambda _{n+1-k +i} \cdot \varphi ) \cdot t^0\bigr ). \end{aligned}$$

Note that $E_n(\varphi )$ does not necessarily live in $\mathcal {A}_{\Phi }[t^{-1}]$. However the composite

$$\begin{aligned} \widehat{E}_n(\varphi ):= ({{\,\textrm{id}\,}}_{\Phi ^{1-n}(A)} \cdot t^{1-n})^n \circ E_n(\varphi ) :A^n \rightarrow \Phi ^{1-n}(A) \end{aligned}$$

does, where for any morphisms $\psi :A \rightarrow B$ in $\mathcal {A}_{\Phi }[t^{-1}]$ we denote by $\psi ^n$ the morphism $\bigoplus _{j=1}^n \psi :A^n = \bigoplus _{j=1}^n A\rightarrow B^n = \bigoplus _{j=1}^n B$ and ${{\,\textrm{id}\,}}_{\Phi ^{1-n}(A)} \cdot t^{1-n}$ is a morphism from $A \rightarrow \Phi ^{1-n}(A)$ in $\mathcal {A}_{\Phi }[t^{-1}]$. So the composite

$$\begin{aligned} F_n(\varphi ):= \widehat{E}_n(\varphi ) \circ U_{\lambda _1,\lambda _2, \ldots , \lambda _n}(\varphi ) :\Phi (A)^n \rightarrow \Phi ^{1-n}(A)^n \end{aligned}$$

of morphisms in $\mathcal {A}_{\Phi }[t^{-1}]$ is given by the matrix

$$\begin{aligned} \begin{pmatrix} {{\,\textrm{id}\,}}_{\Phi ^{1-n}(A)} \cdot t^{-n} &  0 &  \cdots &  0 &  w_1(\varphi ) \\ 0 &  {{\,\textrm{id}\,}}_{\Phi ^{1-n}(A)} \cdot t^{-n} &  \cdots &  0 &  w_2(\varphi ) \\ \vdots &  \vdots &  \ddots &  \vdots &  \vdots \\ 0 &  0 & \cdots &  {{\,\textrm{id}\,}}_{\Phi ^{1-n}(A)} \cdot t^{-n} &  w_{n-1}(\varphi ) \\ 0 &  0 &  \cdots &  0 &  {{\,\textrm{id}\,}}_{\Phi ^{1-n}(A)} \cdot t^{-n} -w_n(\varphi ) \end{pmatrix} \end{aligned}$$

where we define for $k = 1,2, \ldots n$

$$\begin{aligned} w_k(\varphi ) = {{\,\textrm{id}\,}}_{\Phi ^{1-n}(A)} \cdot t^{1-n} \circ v_n = \sum _{i = 0}^{k-1} \lambda _{n+1-k +i} \cdot ({{\,\textrm{id}\,}}_{\Phi ^{1-n}(A)}\cdot t^{i+1-n}) \circ (\Phi ^{-i}(\varphi ^{(i)}) \cdot t^0) \end{aligned}$$

for the morphism $\varphi ^{(i)} = \varphi \circ \Phi (\varphi ) \circ \cdots \circ \Phi ^i(\varphi ) :\Phi ^{i+1}(A) \rightarrow A$ in $\mathcal {A}$.

Note that we get functors of Waldhausen categoriessending an object $(A,\varphi )$ to $\widehat{E}_n(\varphi )$ andsending an object $(A,\varphi )$ to $\widehat{F}_n(\varphi )$. One easily checks using Additivity and suitable exact sequences of $\mathcal {A}_{\Phi }[t,t^{-1}]$- chain complexes that we get the equality of morphisms

$$\begin{aligned} K_s(\widehat{F}_n) = K_s(U_{\lambda _1,\lambda _2, \ldots , \lambda _n}) + K_s(\widehat{E}_n). \end{aligned}$$

Note that $w_n(\varphi ) = 0$ if $\varphi ^{(N)} = 0$, since $\lambda _1 = \lambda _{N-1} = 0$ is assumed. Hence $\widehat{F}_n \circ J$ is given by a lower triangular matrix, all whose entries on the diagonal are given by ${{\,\textrm{id}\,}}_{\Phi ^{1-n}(A)} \cdot t^{-n}$. The matrix $\widehat{E}_n(\varphi )$ is given by a lower triangular matrix, all whose entries on the diagonal are given by ${{\,\textrm{id}\,}}_{\Phi ^{-n}(A)} \cdot t^{-n}$. This implies that the image of both $K_s(\widehat{E}_n) \circ K_s(J)$ and $K_s(\widehat{F}_n) \circ K_s(J)$ lie in the image ofHence the same statement is true for $K_s(U_{\lambda _1,\lambda _2, \ldots , \lambda _n}) \circ K_s(J)$. This finishes the proof that the pairing $T_s$ of (10.7) extends to a continuous pairing

$$\begin{aligned} \widehat{T}_s :W(\Lambda ) \times \overline{K}_s({{\,\textrm{Nil}\,}}(\mathcal {A};\Phi )) \rightarrow \overline{K}_s({{\,\textrm{Nil}\,}}(\mathcal {A};\Phi )) \end{aligned}$$

for $$s \in \mathbb {Z}$$.

Since the image of $\eta$ is dense in *W*(*R*), this continuous extension is unique.

Obviously the pairing $T_n$ turns $\overline{K}_n({{\,\textrm{Nil}\,}}(\mathcal {A};\Phi ))$ into a module over the commutative ring $\overline{K}_0({{\,\textrm{End}\,}}(\Lambda ))$. We conclude from the uniqueness of the extension $\widehat{T}_n$ that it turns $\overline{K}_n({{\,\textrm{Nil}\,}}(\mathcal {A};\Phi ))$ into a module over the commutative ring $W(\Lambda )$.

This finishes the proof of Lemma 10.8. $\square$

### Consequences of the $W(\Lambda )$-module structure on the Nil-terms

#### Theorem 10.11

Let *N* be a natural number. Let $\Lambda$ be a commutative ring with unit $1_{\Lambda }$. Consider an additive $\Lambda$-category $\mathcal {A}$ together with an automorphism $\Phi :\mathcal {A}\xrightarrow {\cong } \mathcal {A}$ of additive $\Lambda$-categories. Let $\mathcal {P}$ be a set of primes. Then we get for every $n \in \mathbb {Z}$:

(i) If $\Lambda$ is an $\mathbb {Z}[\mathcal {P}^{-1}]$-algebra, then $\overline{K}_n({{\,\textrm{Nil}\,}}(\mathcal {A};\Phi ))$ is an $\mathbb {Z}[\mathcal {P}^{-1}]$-module;
(ii) If $\Lambda$ is a $\mathbb {Z}_p$-algebra, then $\overline{K}_n({{\,\textrm{Nil}\,}}(\mathcal {A};\Phi ))$ is a $\mathbb {Z}_p$-module;
(iii) If $N \cdot 1_{\Lambda }$ is zero in $\Lambda$, then $\overline{K}_n({{\,\textrm{Nil}\,}}(\mathcal {A};\Phi ))[1/N]$ vanishes.

#### Proof

Using Lemma 10.8 the proof in Weibel [41, Corollary 3.3] extends to our setting, see also [9, Lemma 3.10]. $\square$

## The Farrell–Jones Conjecture for totally disconnected groups at the prime *p*

The goal of this section is to prove Theorem 1.12. It will follow from Theorem 11.10, which confirms a version of the -Farrell–Jones Conjecture that is more general than the one already treated in [5].

Throughout this section *G* is a *td-group*, i.e., a locally compact second countable totally disconnected topological Hausdorff group.

### Various sets of primes

We need the following sets of primes.

#### Notation 11.1

($\mathcal {P}(G)$) Let *G* be a td-group. Define $\mathcal {P}(G)$ to be the set of primes *q*, for which there exist compact open subgroups $U'$ and *U* of *G* such that $U' \subseteq U$ holds and *q* divides the index $[U:U']$.

If *G* is a compact td-group, then $\mathcal {P}(G)$ is the set of all primes *q* for which there exists a compact open subgroup *U* of *G* such that *q* divides [*G* : *U*]. We have $\mathcal {P}(\widetilde{U}) = \{p\}$ for the compact open subgroup $\widetilde{U} \subseteq Q$ appearing in Assumption 1.9, provided that $\widetilde{U}$ is non-trivial.

#### Lemma 11.2

Let $G'$ be a (not necessarily open or compact) closed subgroup of the td-group *G*. Then $\mathcal {P}(G') \subseteq \mathcal {P}(G)$.

#### Proof

Consider compact open subgroups $$L'$$ and *L* of $$G'$$ satisfying $$L' \subseteq L$$. Since *L* is a compact subgroup of *G*, we can find a compact open subgroup $$L_0 \subseteq G$$ with $$L \subseteq L_0$$, see [5, Lemma 2.3]. Since $$L'$$ is open in *L*, we can find an open subset $$V \subseteq L_0$$ with $$L' = V \cap L$$. Since $$L'$$ is a compact subgroup of $$L_0 $$ and contained in *V*, we can find a compact open subgroup $$L'_0$$ of $$L_0$$ such that $$L' \subseteq L_0' \subseteq V $$ holds, see [5, Lemma 2.3]. Hence we get $$L'_0\cap L = L'$$. This implies that the obvious map $$L/L' \rightarrow L_0/L_0'$$ is injective and hence $$[L:L']$$ divides $$[L_0: L_0']$$. Since $$L_0$$ and $$L_0'$$ are compact open subgroups of *G*, any prime *q* that divides $$[L:L']$$ divides $$[L_0: L_0']$$ and hence belongs to $$\mathcal {P}(G)$$. This implies $$\mathcal {P}(G') \subseteq \mathcal {P}(G)$$. $$\square $$

#### Remark 11.3

Suppose that *G* has up to conjugacy only finitely many maximal compact open subgroups $K_1$, $K_2$, ..., $K_n$. (This assumption is satisfied for every reductive *p*-adic group.) Suppose that Assumption 1.9 is satisfied and let $\widetilde{U}$ be the compact open subgroup appearing in Assumption 1.9. For $i = 1,2, \ldots n$ the subgroup $K_i \cap \widetilde{U}$ has finite index in $K_i$ and we define $\mathcal {P}_i$ to be the finite set consisting of those primes that divide the index $[K_i: (K_i \cap \widetilde{U})]$. One easily checks that $\mathcal {P}(K_i) \subseteq \mathcal {P}(K_i \cap \widetilde{U}) \cup \mathcal {P}_i$ holds. Since $\mathcal {P}(\widetilde{U}) \subseteq \{p\}$, we conclude from Lemma 11.2

$$\begin{aligned} \mathcal {P}(G) \subseteq \bigcup _{i = 1}^n \mathcal {P}(K_i) \subseteq \{p\} \cup \bigcup _{i = 1}^n \mathcal {P}_i. \end{aligned}$$

In particular $\mathcal {P}(G)$ is a finite set.

The notion of a *Hecke category with* *G*-*support* is introduced in [6, Definition 5.1]. Given a Hecke category with *G*-support and a open subgroup $U \subseteq G$, one obtains by restriction to *U* the Hecke category with *U*-support $\mathcal {B}|_U$, see [6, Notation 5.4]. For the notions of a uniform regular or *l*-uniform regular additive category we refer to [4, Definition 6.2].

#### Notation 11.4

($\mathcal {P}(\mathcal {B})$) Let $\mathcal {B}$ be Hecke category with *G*-support. Let $\mathcal {P}(\mathcal {B})$ be the largest set of primes with the property that for any $d \in \mathbb {Z}$ with $d \ge 0$ there is $l(d) \in \mathbb {Z}$ with $l(d) \ge 0$ such that for every compact open subgroup *U* of *G* with $\mathcal {P}(U) \subseteq \mathcal {P}(\mathcal {B})$ the category $(\mathcal {B}_U)_\oplus [\mathbb {Z}^d]$ is *l*(*d*)-uniformly regular coherent.

#### Notation 11.5

($\mathcal {P}(R)$) For a ring *R* let $\mathcal {P}(R)$ be the set of primes which are invertible in *R*.

#### Remark 11.6

If the ring *R* satisfies $\mathbb {Q}\subseteq R$, or, equivalently, that $\mathcal {P}(R)$ consists of all primes, then the Hecke algebra $\mathcal {H}(G,R,\rho ,\omega )$ and the Hecke category with *Q*-support $\mathcal {B}(Q,R,\rho ,\omega )$ are introduced in [7, Section 2.2] and [6, Sections 6.B and 6.C] for any *G*, *N*, ${{\,\textrm{pr}\,}}:G \rightarrow Q$, $\rho$, and $\omega$ as appearing in [7, Section 2.1] and in [6, Section 6.A]. We will assume that $N \subseteq G$ is *locally central*, i.e., its centralizer in *G* is an open subgroup of *G*. If we replace the condition $\mathbb {Q}\subseteq R$ by Assumption 1.9 applied in the case where *G* is replaced by *Q*, then $\mathcal {H}(G,R,\rho ,\omega )$ and $\mathcal {B}(G,R,\rho ,\omega )$ are still defined. Recall that Assumption 1.9 is satisfied if there exists a prime *p* such that *p* is invertible in *R* and *Q* is a subgroup of a reductive *p*-adic group, see [30, Lemma 1.1] and Lemma 11.2.

Now suppose that *R* is uniformly regular and that Assumption 1.9, is satisfied. Then $\mathcal {P}(R)$ is contained in $\mathcal {P}(\mathcal {B}(G,R,\rho ,\omega ))$. The proof of this fact is the same as the one of [7, Theorem 7.2], one just has to observe that [7, Lemma 7.4 (3)] still holds if one replaces the condition $\mathbb {Q}\subseteq R$ by the condition that the order of the finite group |*D*| is invertible in *R*, and that ${{\,\textrm{Idem}\,}}(\mathcal {B}(G,R,\rho ,\omega )_{\oplus })$ is equivalent to ${{\,\textrm{Idem}\,}}(\underline{\mathcal {H}(G,R,\rho ,\omega )}_{\oplus })$, see [6, Lemma 6.6].

Given a set $\mathcal {P}$ of primes, a map of abelian groups is called a $\mathcal {P}$-*isomorphism* if it becomes an isomorphism after inverting every element in $\mathcal {P}$. A map of spectra $\textbf{f}:\textbf{E}\rightarrow \textbf{F}$ is called a *weak *$\mathcal {P}$*-homotopy equivalence* if $\pi _n(F) :\pi _n(\textbf{E}) \rightarrow \pi _n(\textbf{F})$ is a $\mathcal {P}$-isomorphism for all $n \in \mathbb {Z}$. If $\mathcal {P}$ is empty, this is of course the same as a weak homotopy equivalence.

### The Farrell–Jones Conjecture in prime characteristic

The constructions of the following two assembly maps can be found in [5, Definitions 3.8 and 5.10]. Since the following proofs are rather formal, the reader does not need to know the definitions and constructions of , and  and of the following two assembly maps to understand the assertions and proofs of this subsection.

#### Definition 11.7

(*-assembly map*) Let *G* be a td-group and let $\mathcal {B}$ be a Hecke category with *G*-support. The projections $G/U \rightarrow G/G$ induce a map11.8We call this the -*assembly map for* $\mathcal {B}$.

#### Definition 11.9

(${\mathcal {C}\textrm{vcy}}$*-assembly map*) Let *G* be a td-group and let $\mathcal {B}$ be a Hecke category with *G*-support. The maps $P \rightarrow *$ for  induce a map11.9This is the ${\mathcal {C}\textrm{vcy}}$-*assembly map for* $\mathcal {B}$.

We want to prove

#### Theorem 11.10

Let *p* be a prime. Assume that *G* is modulo a compact subgroup isomorphic to a closed subgroup of a reductive *p*-adic group. Let $\mathcal {B}$ be a category with *G*-support.

(i) Let *N* be a natural number such that for the category $\mathcal {B}$ with *G*-support the underlying $\mathbb {Z}$-category $\mathcal {B}$ is obtained by restriction with the projection $\mathbb {Z}\rightarrow \mathbb {Z}/N$ from a $\mathbb {Z}/N$-category $\mathcal {B}'$. Then the -assembly map of (11.8) is a $\mathcal {P}_N$-equivalence for every $n \in \mathbb {Z}$, where $\mathcal {P}_N$ is the set of primes dividing *N*;
(ii) The -assembly map (11.8) is a weak homotopy equivalence, if $\mathcal {P}(G) \subseteq \mathcal {P}(\mathcal {B})$ holds.

If $\mathcal {P}(\mathcal {B})$ consists all primes, then Theorem 11.10 (ii) has already been proved in [5, Theorem 1.11 and Theorem 1.15].

In Sect. 11.3 the proof of Theorem 11.10 will be obtained by inspecting the proof in [5, Theorem 1.11 and Theorem 1.15] taking Theorem 10.11  (iii) into account.

### Reduction from  to ${\mathcal {C}\textrm{vcy}}$

In this subsection we give the proof of Theorem 11.10.

We conclude from by [5, Theorem 5.15],

#### Theorem 11.11

The ${\mathcal {C}\textrm{vcy}}$-assembly map (11.9) is a weak homotopy equivalence for any Hecke category with *G*-support $\mathcal {B}$ if *G* is a reductive *p*-adic group.

So in order to prove Theorem 11.10 in the special case where *G* is a reductive *p*-adic group, we only need to analyse the reduction from ${\mathcal {C}\textrm{vcy}}$ to  which has been carried out in [5, Theorem 14.1] in the case that every prime number lies in $\mathcal {P}(\mathcal {B})$. In the sequel we often omit $\mathcal {B}$ from the notation.

#### Theorem 11.12

Let $\mathcal {P}$ be a (possibly empty) set of primes. Suppose that the ${\mathcal {C}\textrm{vcy}}$-assembly map (11.9)is a $\mathcal {P}$-equivalence and that for every  the canonical mapis a $\mathcal {P}$-equivalence.

Then the -assembly map (11.8)is also $\mathcal {P}$-equivalence.

#### Proof

The arguments on the proof that [5, Theorem 14.7] implies [5, Theorem 14.1] presented in [5, Section 14.B] carry directly over to our setting and lead directly to a proof of Theorem 11.12. Namely, in the diagram appearing [5, Section 14.B] the maps $\alpha _1$ and $\widehat{\alpha _1}$ are $\mathcal {P}$-equivalences and the other four maps labeled by $\simeq$ are also weak homotopy equivalences in our setting. $\square$

Fix $P = (G/V_1,\dots ,G/V_n)$ with $V_i \in {\mathcal {C}\textrm{vcy}}$. Let $K_i \subseteq V_i$ be the maximal compact open subgroup of $V_i$. Set $M:= (G/K_1,\dots ,G/K_n)$. The quotients $\Gamma _i:= V_i / K_i$ are either infinite cyclic or trivial. Let $\Gamma := \Gamma _1 \times \cdots \times \Gamma _n$. Then $\Gamma$ is a finitely generated free abelian group of rank at most *n*. There are canonical maps , sending $\gamma \in \Gamma _i$ to $G/K_i \rightarrow G/K_i, gK_i \mapsto g\widehat{\gamma } K_i$ for any choice $\widehat{\gamma } \in V_i$ which is mapped to $\gamma$ under the projection $V_i \rightarrow \Gamma _i$. These combine to an action of $\Gamma$ on *M* by morphisms in . We write $\underline{\Gamma }$ for the category with exactly one object $*_\Gamma$ whose endomorphisms are given by $\Gamma$. The action of $\Gamma$ on *M* determines a functor  that sends $*_\Gamma$ to $\pi :M \rightarrow P$. Now *h* induces a map , where  is the additive category of twisted Laurent series with respect to the $\Gamma$-action on .

#### Lemma 11.13

Let $\mathcal {P}$ be a set of primes (which may be empty). Then the following assertions are equivalent

(i) For all  the canonical map appearing in Theorem 11.12 is a $\mathcal {P}$-equivalence;
(ii) The canonical map  is a $\mathcal {P}$-equivalence.

#### Proof

This follows from [5, Proposition 14.9] and the commutative diagram [5, 14.10] since the proof of [5, Proposition 14.9], the construction of the commutative diagram [5, 14.10] and the fact that the map (1)–(9) appearing there are weak homotopy equivalences presented in [5, Subsections 14.C, 14.E, and 14.F] carry directly over to our setting, since no assumptions about $\mathcal {B}$ are used. $\square$

#### Lemma 11.14

Let *N* be a natural number such that for the category $\mathcal {B}$ with *G*-support the underlying $\mathbb {Z}$-category $\mathcal {B}$ is obtained by restriction with the projection $\mathbb {Z}\rightarrow \mathbb {Z}/N$ from a $\mathbb {Z}/N$-category $\mathcal {B}'$. Then theis a $\mathcal {P}$-homotopy equivalence for $\mathcal {P}$ the set of primes dividing *N*.

#### Proof

By inspecting the definitions the additive category  inherits the structure of an additive $\mathbb {Z}/N$-category. Now one proceeds by induction over the rank for $\Gamma$ using the isomorphism (4.5) and Theorem 10.11  (iii). $\square$

#### Lemma 11.15

If $\mathcal {P}(G) \subseteq \mathcal {P}(\mathcal {B})$ holds, then then canonical mapis a weak homotopy equivalence.

#### Proof

The proof that the map appearing in Lemma 11.15 is a weak homotopy equivalence is a variation of the one appearing in [5, Section 14.H]. By the arguments in [5, Section 14.H] it suffices to check that the map [5, (14.14)] is a weak homotopy equivalence, since the map appearing in Lemma 11.15 is the map (7) appearing at the very bottom of the diagram [5, (14.10)]. Hence it suffices to check that the argument appearing [5, Lemma 14.16] carries over if we assume $\mathcal {P}(G) \subseteq \mathcal {P}(B)$ and do not use not the stronger [5, Assumption 3.11], which is equivalent to requiring that we can choose $\mathcal {P}(\mathcal {B})$ to be the set of all primes, since then we can apply [4, Theorem. 14.1], exactly as we did in [5, Section 14.H] after the proof of [5, Lemma 14.16].

Let $(U_{r,i})_{r = 1,\ldots ,n, i \in \mathbb {N}_{\ge 1}}$ be the system of compact open subgroups of *G* as they appear in [5, Section 14.H]. In view of the proof of [5, Lemma 14.16], it remains to explain why for every $i \in \{1,2, \ldots , n \}$ and every element $\lambda \in |Q_i|$ the category $\bigl ((\mathcal {B}|_{G_{\lambda }})_\oplus \bigr )[\mathbb {Z}^d]$ is *l*-uniformly regular coherent, where $|Q_i| = G/U_{1,i} \times \cdots \times G/U_{n,i}$. In the proof of [5, Lemma 14.16] we had used [5, Assumption 3.11] precisely at this place, but nowhere else in the proof of [5, Theorem 14.7], and we do not want to use this assumption here. In the situation here it suffices to show $\mathcal {P}(G_{\lambda }) \subseteq \mathcal {P}(\mathcal {B})$. This follows from the assumption $\mathcal {P}(G) \subseteq \mathcal {P}(\mathcal {B})$ and Lemma 11.2. This finishes the proof of Lemma 11.15. $\square$

#### Proof of Theorem 11.10

Theorem 11.10 follows from Theorems 11.11, 11.12, Lemmas 11.13, 11.14, and 11.15, provided that *G* is a reductive *p*-adic group. Now the general case, where *G* is modulo a compact subgroup isomorphic to a closed subgroup of a reductive *p*-adic group, follows from the proof of [6, Theorem 1.5], which directly carries over to our setting. $\square$

We state the following version of the -Farrell–Jones Conjecture.

#### Notation 11.16

($\mathcal {P}(\mathcal {B},G)$) Let $\mathcal {B}$ be Hecke category with *G*-support. Define $\mathcal {P}(\mathcal {B},G)$ to be the set of primes *q* that belong to $\mathcal {P}(G)$ but not to $\mathcal {P}(\mathcal {B})$.

#### Conjecture 11.17

((Generalized) -Farrell–Jones Conjecture) The td-group *G* satisfies the *(Generalized)* *-Farrell–Jones Conjecture*, if for every Hecke category with *G*-support the -assembly map (11.8)is a $\mathcal {P}(\mathcal {B},G)$-equivalence.

If $\mathcal {P}(G) \subseteq \mathcal {P}(\mathcal {B})$, then Theorem 11.10 (ii) confirms Conjecture 11.17 if *G* is modulo a compact subgroup isomorphic to a closed subgroup of a reductive *p*-adic group.

### Proof of Theorem 1.12

#### Proof of Theorem 1.12

(i) We conclude from Lemma 11.14 that the canonical mapis a $\mathcal {P}_N$-homotopy equivalence for $\mathcal {P}_N$ the set of primes dividing *N* provided that *N* is a natural number such that for the category $\mathcal {B}$ with *G*-support the underlying $\mathbb {Z}$-category $\mathcal {B}$ is obtained by restriction with the projection $\mathbb {Z}\rightarrow \mathbb {Z}/N$ from a $\mathbb {Z}/N$-category $\mathcal {B}'$.

Assertion (i) of Theorem 1.12 follows from Theorem 11.10 (ii), as for the specific choice of $\mathcal {B}= \mathcal {B}(G,R,\rho ,\omega )$ appearing in Remark 11.6 the map assembly (1.10) is obtained from the -assembly map (11.8) by applying $\pi _n$, see [6, Section 6.D] and $\mathcal {B}(G,R,\rho ,\omega )$ has the required property above because of the assumption $N \cdot 1_R = 0$.

(ii) In view of the arguments appearing in the proof of [8, Theorem 2.16] based on the equivariant Atyiah-Hirzebruch spectra sequence of [8, Theorem 2.1], it suffices to show that $K_n(\mathcal {H}(p^{-1}(U),R,\rho |_{p^{-1}(U)},\omega )) \cong H_n^G(G/U;\textbf{K}_{\mathcal {B}(G,R,\rho ;\omega )})$ vanishes for every $n \le -1$ and every compact open subgroup $U \subseteq Q$. The arguments in the proof of [7, Lemma 8.1] apply also to our setting and imply that it suffices to show for any compact normal subgroup $K \subseteq p^{-1}(U)$ with $K \in P$ that $K_n(\mathcal {H}(p^{-1}(U)//K,R,\rho |_{p^{-1}(U)},\omega )) = 0$ holds for $n \le -1$ The proof of [7, Lemma 7.5] carries over to our setting and implies that $\mathcal {H}(p^{-1}(U)//K,R,\rho |_{p^{-1}(U)},\omega )$ can be identified as a crossed product ring $R *D$ for some group *D* invertible in *R*. As *R* is Artinian, $R *D$ and hence are $\mathcal {H}(p^{-1}(U)//K,R,\rho |_{p^{-1}(U)},\omega )$ are Artinian. Now we conclude from [25, Theorem 4.15 (ii)] applied in the case of a trivial group and $k = 0$ that $K_n(\mathcal {H}(p^{-1}(U)//K,R,\rho |_{p^{-1}(U)},\omega )) = 0$ holds for $n \le -1$. This finishes the proof of assertion (ii). $K \subseteq Q$.

(iii) The proof that the assembly (1.10) is bijective is analogous to the proof of assertion (i) but now using Lemma 11.15 instead of Lemma 11.14. $\square$

#### Remark 11.18

It is conceivable that assertion (ii) appearing in Theorem 1.12 is still true if we do not invert *N*. (We have no proof.) For instance it may be true that, for a prime *p*, an Artinian ring *R* for which *p* is invertible in *R*, and a subgroup *G* of a reductive *p*-adic group,

$$\begin{aligned} K_n \big (\mathcal {H}(G;R)\big ) = 0 \quad \text {for}\; n \le -1 \end{aligned}$$

holds and the mapis bijective, or, equivalently, that the assembly map (1.10) is bijective for $n \le 0$. (The latter is not true in general under the assumptions above for $n \ge 1$.)
